# Microfluidics for Blood Disorders and Hematological Disease Monitoring and Modeling

**DOI:** 10.3390/ijms27104581

**Published:** 2026-05-20

**Authors:** Mengjia Hu, Nathan Henderson, Steven A. Soper, Malgorzata A. Witek

**Affiliations:** 1 Department of Chemistry, The University of Kansas, Lawrence, KS 66045, USA; m367h922@kumc.edu (M.H.); nhenderson4@kumc.edu (N.H.); 2 Center of BioModular Multiscale Systems for Precision Medicine, The University of Kansas, Lawrence, KS 66045, USA; 3 Kansas Institute for Precision Medicine, University of Kansas Medical Center, Kansas City, KS 66160, USA; 4 Department of Cancer Biology, University of Kansas Medical Center, Kansas City, KS 66160, USA; 5 Bioengineering Program, The University of Kansas, Lawrence, KS 66045, USA; 6 Department of Mechanical Engineering, The University of Kansas, Lawrence, KS 66045, USA

**Keywords:** microfluidic, lab-on-a-chip, blood disorders, liquid biopsies, organ-on-a-chip

## Abstract

Blood disorders encompass a wide range of diseases including anemia, hemophilia, thrombotic disorders, platelet dysfunction, and hematological cancers, making blood disorders a major global health concern. These conditions can impair processes vital to human physiology including oxygenation, coagulation, and immune defense. Hematologic malignancies, both chronic and acute, require timely diagnosis and ongoing disease monitoring for effective clinical management. Microfluidic technologies have emerged as promising alternatives to benchtop techniques for diagnosing and monitoring hematological disorders. For example, microfluidic assays can be used for the isolation and characterization of liquid biopsy markers such as rare cells, extracellular vesicles, and cell-free molecules to support disease management in a minimally invasive manner while the process automation afforded by microfluidics decentralizes healthcare, making it more accessible. Advances in lab-on-a-chip technologies, including large-scale fabrication methods and novel design strategies, will provide tools for the clinical validation of biomarkers and the translation of these technologies from the laboratory bench to the patient bedside. In this review, we will show that microfluidic devices enable disease monitoring via high-throughput analysis of liquid biopsy samples for the detection of rare disease-specific biomarkers found in blood, plasma, urine, etc., providing an alternative to standard benchtop testing using specimens secured via invasive bone marrow procedures, typically used for managing blood-based diseases. A key advantage of microfluidics is their ability to manipulate blood components at scales that closely mimic the body’s microvascular environment. Not surprisingly, microfluidic vascular models have been developed to replicate physiological rheology enabling quantitative assessment of blood cell deformability, aggregation, or clot formation. We provide a critical perspective on the use of the microfluidic “organ-on-chip” designed for blood disorders’ modeling and employed to recapitulate the blood cancer microenvironment. A summary of advances in microfluidic strategies for detection, diagnosis, drug screening, and mechanistic investigations of blood disorders, and future directions for precision testing, will be presented.

## 1. Introduction

Blood diseases have a significant impact on human health. affecting life quality and longevity. Non-cancerous blood disorders are the conditions that affect blood cells and platelets and cause issues related to blood clotting with potentially profound consequences. Abnormal clotting increases the risk of deep vein thrombosis, pulmonary embolisms, stroke, or heart attack. Malignant blood diseases affect blood cell production and as a result disturb the function and concentration of normal blood cells. Regardless of the severity of the blood disorder, patients require frequent testing to assess disease status and follow up with the appropriate medication and treatments to either keep the disease in a chronic state or prevent life-threatening conditions. Blood and bone marrow (BM) disorders are diagnosed when symptoms suggest abnormal levels of certain cells or the presence of abnormal blood cells. Blood diseases affect the components of BM and blood, such as red blood cells (RBCs) [1], white blood cells (WBCs) [2], platelets [3], hemoglobin (Hb) [4], vascular endothelium [5], or plasma proteins. Genetic factors [6,7,8], nutritional deficiencies [9,10], infections, and autoimmune disorders [11,12] are recognized causes of many blood disorders. Throughout this review, “monitoring of disease” encompasses the assessment of its status or burden via minimal residual disease (MRD) analysis, while “modeling” focuses on understanding the underlying dynamic and kinetic processes that drive disorder or disease onset and evolution.

### 1.1. Blood Disorders and Cell Characteristics—Role of Microfluidics

Common blood disorders include anemia, hemophilia, thrombosis, and hematological cancers such as leukemia, lymphoma, and myeloma [13,14]. These conditions affect cells’ concentration and morphology/mechanical properties (e.g., shape, size, circularity, or volume). For example, normal mean corpuscular volume (MCV), which expresses the average size of RBCs, is typically ~100 fL. Individuals with macrocytosis have ~15% larger MCV. In poikilocytosis, the RBC shape differs from the standard biconcave disk, which may affect their ability to carry oxygen. Hereditary elliptocytosis, an inherited group of RBC membrane disorders, causes elliptical, oval, or elongated cells, which leads to reduced cell elasticity [15]. Sickle cell disease (SCD) is characterized by sickle-shaped RBCs with reduced deformability and elevated intracellular viscosity [15]. The culprit of malaria, parasite *Plasmodium falciparum*, alters the RBC membrane mechanical properties by inserting its proteins into the membrane, leading to the loss of cell shape and deformability.

Platelet size can be affected by medication. As an example, treatment with thrombopoietin increases stimulation by cytokines [16]. The normal mean platelet volume is 5–10 fL [17] and changes in this may reflect the maturation stage of the platelet. Platelets with volumes >12 fL are referred to as “large,” and their abundance varies between 18% and 48% of all platelets [16]. Some studies indicate that large volume platelets can lead to a higher risk of thromboembolism [18].

Leukemia and lymphoma cells also possess distinct biophysical properties compared to their healthy counterparts. For example, myeloid lineage cancer cells are rigid; they have been shown to be 18× less flexible than lymphoid cancer cells and 6× less flexible than normal neutrophils [19]. Clearly, blood disorders are characterized not only by the abnormal abundance of cells [20,21,22,23,24,25,26,27,28] (Table 1), but also by the biomechanical properties that can cause additional complications such as microvascular obstructions, hypoxia, and organ damage [29].

Analytical and surface characterization methods such as atomic force microscopy (AFM) [37,38], micropipette aspiration [39], and optical/magnetic tweezers [40,41,42,43] can be used to elucidate cells’ mechanical properties (e.g., Young’s modulus, membrane stiffness, viscoelasticity, and cell volume). However, they operate at low throughput (few cells/h) with cells attached to a substrate to lock them into a two-dimensional (2D) format. In contrast, microfluidic devices offer advantages for measuring the biomechanical properties of cells in a three-dimensional (3D) environment and under flow conditions supplying gradient concentration of media/stimuli with shear forces that resemble physiological conditions. Microchannel geometries can be engineered to provide channel constrictions for deformability assays [44], to allow analysis of multiple single cells simultaneously providing high throughput. Owing to the small internal volumes of microfluidics, rare cells can be analyzed without loss and, due to the flow-through formats of microfluidics, cells can be collected and tested in other downstream assays. Further advantages of streamlining cell analysis or sorting based on cell mechanics [45] can be accomplished by merging analytical methods with microfluidics (i.e., optical tweezers with microfluidics [46]).

### 1.2. Need for Screening and Diagnosing Hematological Disorders and Diseases

**Figure 1:**
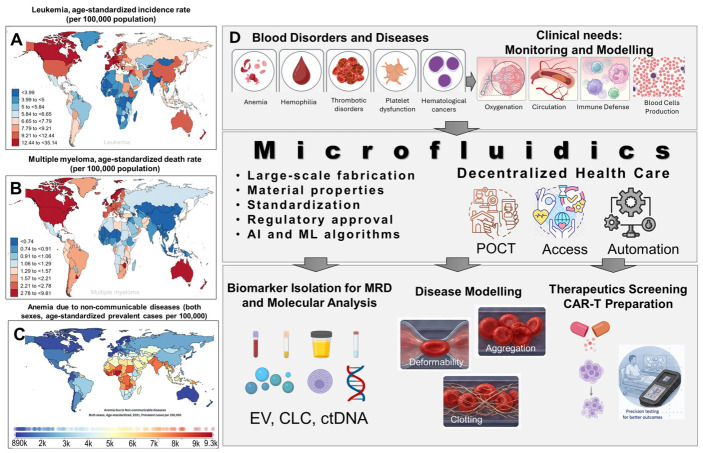
Global heat maps for: (**A**) Age-standardized incidence rate of leukemia in 2019 [47], reproduced from Zhang et al. (2023). Creative Commons CC BY 4.0 license. (**B**) Age-standardized death rate of MM in 2019 [47], reproduced from Zhang et al. (2023). Creative Commons CC BY 4.0 license. (**C**) Age-standardized rate of anemia in 2021 [49], reproduced from Yu et al. (2025). Creative Commons Attribution License CC BY. (**D**) A schematic representation of the topics covered in the review. Created in PowerPoint with elements from BioRender and ChatGPT (Version GPT-4o). Henderson. N. (accessed on 4 May 2026).

Hematological malignancies caused by the disruption of normal hematopoietic cells’ function are one of the most common cancers [47]. Their risk varies with age, gender, and geographical location, which links them to genetic and environmental risk factors. Acute leukemias represent a substantial global health burden with persistently high incidence rates [48]. In 2019, acute myeloid leukemia (AML), acute lymphoid leukemia (ALL), chronic myeloid leukemia (CML), chronic lymphoid leukemia (CLL), multiple myeloma (MM), and plasma cell leukemia (PCL) had the highest reported age-standardized incidence or death rates in Western Europe, North America, and Australasia (Figure 1A,B) [47]. The global burden of anemia caused by non-communicable diseases (i.e., diabetes, cardiovascular and kidney disease) increased 48% between 1990 and 2021, affecting 3.7 billion people, as seen in Figure 1C [49], with numbers predicted to double by 2045 for the female population [49].

The likelihood of a particular blood cancer changes with age; ALL occurs primarily in children, while AML is diagnosed equally frequently in children and adults, whereas CLL and CML mainly arise in adults. Due to an aging global population, the incidence of AML is projected to increase by 2040 [50], and global trends also suggest increasing incidence of non-Hodgkin lymphoma (NHL) and Hodgkin lymphoma (HL) [48]. The diagnosis and treatment of some subtypes of leukemia have improved greatly in high-income countries; for example, a 5-year survival rate of pediatric ALL is 90%, but it is only 58% in low resource areas [51].

The prevalence of RBC disorders is high in low- and middle-income countries, and unfortunately, testing rates remain low due to cost and limited diagnostic infrastructure [52]. Hb disorders, such as SCD or thalassemia, are estimated to affect >0.5 million infants/year [53]. Early diagnosis of SCD in newborns is critical for designing a plan for lifelong health challenges and, in general, all SCD patients should be frequently screened for impending sickle cell crises. Unfortunately, access to simple diagnostic tests that can identify preventable complications is limited [54,55].

Early diagnosis of blood disorders and ongoing disease monitoring are critically important for patient benefits but also can lead to lower cost of medical care. The development of inexpensive, rapid testing modalities for screening and diagnosing RBC, WBC, or platelet disorders could be of utmost importance in the blood banking field, where screening donors and blood eligibility is required. Considering the Red Cross statistics (https://www.redcrossblood.org), each year ~7 million people in the U.S. alone donate blood and all donations are tested for infectious diseases (i.e., viral and bacterial contaminations, and recently malaria to avoid transfusion-transmitted diseases [56]).

## 2. Can Microfluidics Service Blood Disorders in the Clinic?

Microfluidic-based technologies have emerged as novel tools for providing information on disease burden and disease molecular makeup, bridging the gap between the research bench and the patient bedside (i.e., bench-to-beside transition). It is notable that these devices are being integrated into fluidic systems to perform multi-step analysis and provide full process automation allowing for better delivery of testing scenarios to geographical areas where benchtop testing can be problematic due to the need for highly trained operators and the need for specialized equipment [57,58,59,60,61,62,63].

This review summarizes advances in the use of microfluidics in clinical applications specifically for the diagnosis, screening, and monitoring of various blood disorders. We will review cell sorting [64,65], single-cell analysis [66,67], rare cell enrichment [60,68,69,70,71,72,73,74,75,76,77], exosomes or extracellular vesicle (EV) isolation [78,79,80,81], molecular testing [82,83,84,85,86,87], organ-on-a-chip models [88,89,90,91,92,93], and drug screening [94,95,96,97,98] platforms used for specifically managing patients with blood disorders. We highlight lab-on-a-chip technologies that offer advantages related to low reagent consumption, full automation, high-throughput capabilities, cost-effectiveness, and portability for potential point-of-care testing (POCT) [78,79,80,99,100,101,102]. Moreover, we will discuss vascular-on-chip models that can recapitulate cells’ normal microenvironment to allow for fundamental studies in areas such as cell–cell interactions, drug screening, immunotherapy, and response monitoring [90,91,92,93]. We conclude with future directions of microfluidics for precision testing of hematological disorders.

### 2.1. Microfluidics—Choice of Material, Fabrication Strategy, and Commercialization

The choice of material used to construct a clinical or modeling device is dictated by the requirements of the application (cell-based assay vs. molecular biology test), the readout modality (electrical vs. optical), the fabrication strategy at volumes sufficient to support the required throughput, prototyping capabilities, and the ability to reproducibly and stably activate or modify the device’s internal surface. Key material properties relevant to biological assays and the associated fabrication methods are listed in Table 2.

Material gas permeability is an important factor in supporting cell viability during cell modeling experiments. Polydimethylsiloxane (PDMS) is a good choice in these applications because its high gas permeability allows the passive and continuous exchange of O_2_ and CO_2_ without active supplementation of the culture media. Flexdym elastomer has been used for cell culture and sensors [103]. Thermoplastics can also support cell viability; however, owing to a lower permeability barrier to gases, the culture media must be actively supplied with O_2_/CO_2_. In contrast, thermoplastics offer a significant advantage over PDMS as moisture barriers, reducing evaporative losses and supporting longer-duration experiments.

For assays involving fluorescence readout or drug testing, Cyclic Olefin Copolymer (COC) and Cyclic Olefin Polymer (COP) thermoplastics are an attractive alternative to glass or PDMS. Their low autofluorescence reduces background signal interference, and their minimal drug absorption prevents compound loss and avoids confounding pharmacological results. PDMS is known to absorb small hydrophobic molecules and drugs.

Whenever optical clarity and chemical inertness are important, all solid substrates can serve this purpose efficiently. Thin cover plates support high-resolution imaging with low background signals, and its chemically inert surface prevents undesired interactions with biological samples. The inherent elasticity of PDMS makes it well suited for biomimetic microvascular models, where the mechanical compliance of the device channels should replicate the behavior of native blood vessels or tissue. However, elastomeric thermoplastics may suit this purpose as well.

Noteworthy reviews and commentaries have been published covering the topic of microfluidic manufacturing and their transition to commercial and clinical venues [104,105,106,107,108,109]. The commercialization of devices requires large-scale production, which can be realized by injection molding. Injection molding is typically used for thermoplastics. However, it can be used for manufacturing PDMS devices as well. Filion et al. demonstrated that producing PDMS devices through injection molding yields reproducible parts in high volume and enables the mass-manufacture of biomedical microfluidic devices [110]. While 3D printing allows for fast and inexpensive prototyping, currently it cannot compete with injection molding in terms of high-scale production at low cost [108].

Although microfluidic devices themselves are often low-cost and portable, many microfluidic testing strategies require laboratory equipment and trained personnel for sample pre- and post-processing [111]. Efforts to address this have led to the development of fully integrated systems that incorporate on-chip sample preparation devices [112], as well as the coupling of microfluidic platforms with smartphones and other widely available consumer electronics to enable on-site analysis and the transmission of results to healthcare decision makers [113,114]. Irrespective of the level of innovation in biomedical micro-devices and systems as well as the potential for mass production, commercialization still represents a challenge [16].

The technical complexity of some microfluidic systems, combined with the logistical and financial burden of the large-scale clinical trials required for clinical validation to meet regulatory requirements, creates barriers to market entry. Navigating these barriers requires coordinated effort across disciplines, bringing together engineers, biologists, clinicians, and business leaders to align device design, clinical utility, and targeting viable routes to market to realize translation. However, the number of microfluidic-based devices submitted to the FDA for evaluation and regulatory assessment is increasing in step with the growth of the microfluidic industry [115].

It is important to move beyond proof-of-concept experiments suitable for small-scale production and into a design concept with more scalable solutions that are more likely to translate into viable platforms for in vitro diagnostics (IVD). This can be realized by implementing more inter-device standardization frameworks. Standardization can simplify the evaluation process for regulators and provide a consistent evidential framework for assessment. On the development side, the design strategy should create opportunities to engage multiple institutional partners in multi-site validation studies, demonstrating that clinical performance is universal and not an artifact of the originating laboratory. A persistent challenge in securing regulatory approval, however, lies in the field’s difficulty in articulating the commercial and public health case for a new device, which requires identifying the actual market value of the technology, demonstrating unmet clinical need, and ways in which the proposed device outperforms existing solutions. Ultimately, the question regulators and customers alike will ask is whether the benefit to the end user is compelling enough to justify switching from established products to those that are microfluidic-based.

### 2.2. Peripheral Blood (PB) and Liquid Biopsies as an Alternative to Bone Marrow Biopsies (BMBs)

**Figure 2:**
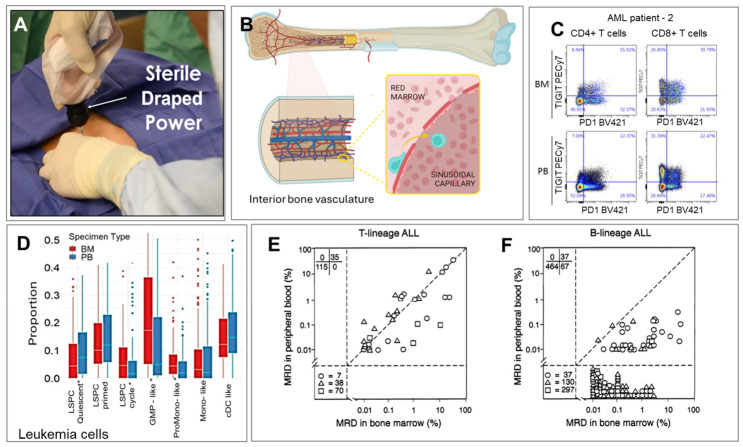
(**A**) BMA collection procedure [116], reproduced from Chahla et al. (2017). Creative Commons CC-BY-NC-ND. (**B**) Structural organization of BM and the associated interwoven vascular system. Created in Biorender. Henderson. N (2026) https://BioRender.com/9jat77s, accessed on 4 May 2026. (**C**) MFC characterization of cells collected from paired peripheral blood (PB) and bone marrow (BM) samples. Expression of checkpoint receptors on AML cells in PB and BM was not statistically different [124], reproduced with permission from Lesley et al. (2017). Copyright Elsevier, 2017. (**D**) Composition of AML cells in BM and PB as determined by CIBERSORTx [125] which identifies a cell type from RNA expression data. Results demonstrated correlation of leukemic stem and progenitor cells (LSPCs) in BM and PB. Statistically different proportion of cells between BM and PB marked with * [126], reproduced from Caliskan et al. (2024). Creative Commons CC BY 4.0 license. (**E**) Levels of leukemia cells in paired BM and PB samples from T-ALL patients analyzed by immunophenotyping with a T cell marker and TdT [127], reproduced with permission from Coustan-Smith et al. (2002). Copyright Elsevier, 2002. (**F**) Levels of pre-B-ALL cells in paired BM and PB samples analyzed by qPCR using Ig/TCR gene rearrangements [127], reproduced with permission from Coustan-Smith et al. (2002). Copyright Elsevier, 2002.

Some disorders can be assessed directly from peripheral blood (PB) through a minimally invasive blood draw, which is particularly attractive for facilities that lack infrastructure to secure bone marrow aspirates (BMAs), which usually consists of invasively securing the BMA during a bone marrow biopsy (BMB), Figure 2A [116]. This highly invasive procedure requires a hospital setting and involves highly trained medical personnel [117], making it expensive and infeasible for frequent testing. Other challenges related to BMAs include hemodiluted samples, low to no BMA collection, and misdiagnoses caused by sampling errors [117,118,119]. There are also several consequences associated with BMB including bleeding and coagulation [120,121,122,123].

Imaging methods such as magnetic resonance imaging (MRI), computer tomography (CT), and Positron Emission Tomography (PET) are complimentary to BM sampling when assessment of the disease status in MM patients is needed. While informative, the interpretation of MRI results can be complicated due to natural bone changes in older patients or variations related to bone necrosis but not necessarily MM as an example [128]. The disadvantage of PET is its limited spatial resolution and the fact that it exposes patients to high radiation and therefore is not suitable for frequent testing [129,130]. CT is routinely used in clinical practice for the assessment of the extent of MM when MRI is not available; however, results may appear inconclusive as guidelines suggest that if no signs of bone changes are detected on CT, MRI should be performed [131]. Access to MRI machines is not guaranteed in every hospital and the test requires a dedicated technologist and radiologist to interpret the results. While imaging methods for treatment efficacy may be useful, if disease progression is detected no molecular information on actionable targets is gained to guide further treatment decisions.

These examples underscore the need for new technologies to address current inefficiencies for managing blood diseases. A growing body of literature highlights the urgent need for novel strategies to improve the early detection, diagnosis, and management of these diseases using PB [48]. Since liquid biopsies can be obtained from a wide variety of biological fluids via a minimally invasive way, prognostic or diagnostic biomarkers can be evaluated frequently, providing near-real-time information on disease status and treatment response [127,132,133,134,135,136].

The structural organization of BM tissue is interwoven with the vascular system [31], with the central artery splitting into smaller ones forming a dense network of arterioles extending into venous vessels (Figure 2B). The vessels merge with sinuses that drain into PB while periosteal arteries penetrate the bone and merge with the BM [31]. While leukemias originate in BM, leukemic cancer cells are found in PB owing to the migration of cancer cells from BM. The mechanism of cancer cell transport to PB may involve both elevated pressure in the BM, changes in vascular permeability, leukemic cells’ ability to modify the chemical signals that are designed to retain immature cells in the BM, or by disrupting hematopoiesis and BM architecture [137]. Similarly in lymphomas that originate in the lymphatic system, cancer cells spread to other lymph nodes and organs including BM, and in advanced aggressive disease, lymphoma cells can also be found in PB [137,138].

Regardless of the route of cancer cell transport from BM or lymph nodes to PB, circulating leukemia cells (CLCs) and other cancer signatures can be detected in PB (Figure 2C–F) at varying levels mainly due to the limits of detection (LODs) of the assay used for detection [31]. The hypothesis arises: do cancer cells reside in PB but are non-quantifiable because testing methods lack analytical sensitivity? The review of the literature supports this supposition.

For example, in the case of MM, cfDNA and circulating MM, cells have shown similar somatic mutations and copy number alterations following an analysis of these liquid biopsy markers as secured from BMA samples [139]. NHL originating in lymph nodes disseminated circulating cells to both BM [32] and PB [140,141], as cancer cells were detected in 45% of patients diagnosed with advanced NHL disease, but not in patients with early-stage disease [141]. The detection of NHL cancer cell signatures required testing of immunoglobulin (Ig) and T-cell receptor gene rearrangements (Ig/TR) using Southern blots (i.e., DNA hybridization). This method requires 10^6^ cells for analysis and can detect abnormal cells only at the 1% level [142]. In classical HL, cancer cell signatures were detected in PB using PCR and next-generation sequencing (NGS), as well as multiparameter flow cytometry (MFC) [143,144]. In active diseases, AML CLCs were found in PB using MFC with the ability to detect one abnormal cell in the presence of 10,000 normal cells. The immunophenotype of PB cells was representative of the BM’s AML cells (Figure 2C) [124]. A method called CIBERSORTx [125] identified AML cell-type-specific transcripts in PB using an algorithm that infers cell type from mRNA profiles (Figure 2D) [126].

The detection of T-cell ALL (T-ALL) CLCs in BMAs and PB showed >85% correlation (Figure 2E) and furthermore, the frequency of leukemia cells in BMAs and PB agreed even >3 months post treatment [127,145]. Unlike T-ALL, the abundance of pro-B-cell ALL (B-ALL) CLCs in PB was lower than in BM as determined by Ig/TR gene rearrangements serving as “DNA fingerprints” of the leukemic B-ALL cells (Figure 2F) [127,145]. Ig/TR RT-qPCR can detect B-ALL cells in >95% of patients; however, while the assay can find 10^−4^ to 10^−5^ CLCs per normal blood cell count it requires NGS of Ig/TR rearrangements for each patient to design patient-specific PCR primers [146]. Brisco et al. confirmed B-ALL CLC detection from 2 mL of PB when the level of cancer cells in BM was >10^–4^ [147].

In summary, the abundance of liquid biopsy markers in PB will dictate the LOD requirements of the analytical method because the abundance of disease-associated markers in PB is lower than that found in a BMA. The identification of disease signatures from PB compared to BMAs offers numerous benefits: (i) better specificity owing to the absence of normal myeloid progenitor cells or hematogones in PB that can express similar cancer cell antigens [148]; (ii) easier access to the testing sample secured via a minimally invasive procedure; (iii) more frequent testing; and (iv) the early identification of impending acute disease. However, any downstream molecular determinations must be preceded by an enrichment and purification step to select the diseased cells, which are a vast minority in a mixed population, from normal blood cells.

The NGS-based FDA-approved ClonoSEQ^®^ assay detects MRD in MM, ALL, and CLL based on the presence of unique disease-associated cancer cell immunoglobulin sequences such as IgH, IgK, and IgL rearrangements and IgH-BCL1/2 translocations, and the sequencing of DNA fragments. The assay involves the isolation and amplification of gDNA from BMAs or PB depending on the disease, and the sequencing of B and T cells’ variable, diversity and joining gene segments (VDJ) or VJ regions [149]. The assay’s success depends on securing baseline diagnostic samples, typically from BMA, to identify disease-associated VDJ or VJ sequences that are used as a blueprint for subsequent MRD monitoring. The DNA sequence’s uniqueness is assessed and confirmed by searching databases of Ig rearrangements that must not be identified in healthy cells. Low distinctiveness between VDJ or VJ sequences makes MRD testing using this strategy impossible. Ching et al. [149] reported that 4% of patients (ALL, CLL, and MM) were lacking uniquely rearranged Ig. Pulsipher et al. [150] reported 12% B-ALL patients with noninformative sequences.

For the quantification of the total nucleated cells in a sample to determine MRD status, diploid copies of the genomic regions (i.e., normal gDNA) are amplified and sequenced as well [149]. The library is sequenced ~10× per clone, and the tumor Ig clones are defined as those present >5% of the sequence counts [150]. The ClonoSEQ demonstrated a LOD of ~2 malignant cells for DNA input levels ranging between 200 ng and 40 μg, detecting MRD ~ 10^−6^ of the total nucleated cell population. The reported precision at LOD was 68% due to experimental error and in some part the handling of a small number of cells governed by Poisson distribution. The variability for 612 cancer cells was 18% [149].

### 2.3. Cell-Based Microfluidics for Minimal Residual Disease (MRD) Detection for Leukemias

Hematologic malignancies are classified into three major types based on the specific type of blood cells involved: leukemia (myeloid or lymphoid cells); lymphoma (lymphocytes); and myeloma (plasma cells) [151]. Blood cancers are a result of the uncontrolled proliferation and abnormal differentiation of blood cells or lymphoid cells leading to disrupted immune and BM function (Table 1) [152,153].

**Figure 3:**
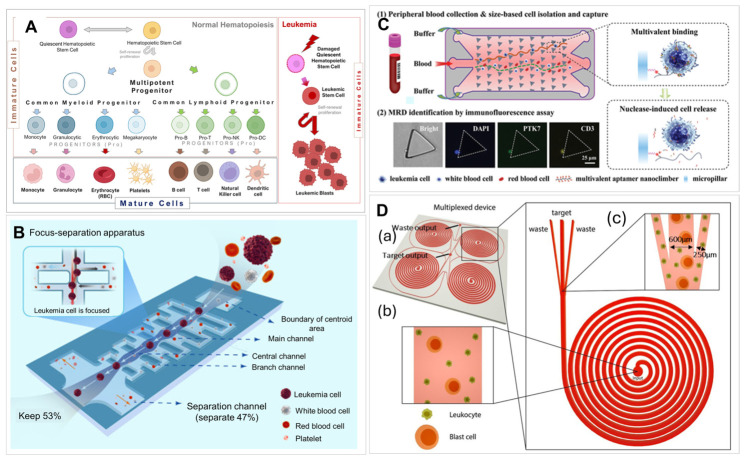
(**A**) An illustration of the process of hematopoiesis in BM and hematological cancer development. A scheme of blood compositions and blood cell development. Created in BioRender. Henderson. N. (2026) https://BioRender.com/mmfdgi5, accessed on 4 May 2026. (**B**) A schematic and conceptual design of a microfilter device designed for CLC enrichment [180], reproduced from Cai et al. (2023). Creative Commons CC-BY-NC-ND. (**C**) A DLD device modified with a multivalent aptamer nanoclimber for the capture and release of leukemia cells and the immunofluorescence-based identification of CLCs [169], reproduced with permission from Liu et al. (2020). Copyrights John Wiley and Sons, 2020. (**D**) A schematic showing the workflow and concept of an inertial-based microfluidic device that uses the known physical properties of cells to sort cells by physical attributes, such as size. (**a**) A 3D overview of the multiplexed CLC biochip; (**b**) a close-up view of the unsorted sample distribution at the input position; (**c**) a close-up view of the sorted sample distribution at the bifurcation point [181], reproduced from Khoo et al. (2019). Creative Commons CC BY 4.0 license.

Leukemia is a group of blood cancers that begin in the abnormal proliferation of hematopoietic stem cells in the BM that lead to the formation of numerous abnormal WBCs (Figure 3A) [152,153]. Major subtypes of leukemia include ALL, AML, CLL, and CML, which can be characterized by their distinct genetic and immunophenotypic profiles [153]. Leukemia affects BM and disrupts normal blood cell production and immune functions.

Accurate MRD detection is the most important prognostic indicator for blood cancers [140,146,154,155] as it is used for the prediction of risk of relapse and guidance on therapeutic strategies [156]. Blood smear-based analyses are subjective, time-consuming, and cannot confidently detect low-abundance leukemia cells. Other MRD detection methods used are MFC, PCR, and NGS [74,156,157]. MFC can achieve a detection limit of ~1 leukemia cell among 10,000 WBCs (10^−4^ or 0.01%), with the success of the analysis dependent on the number of cells analyzed, gating strategy employed, experience of the MFC operator, and the number of markers used [157]. Molecular PCR techniques for MRD detection target known leukemia aberrations or mutations, such as *BCR-ABL1* in ALL and CML; *NPM1* insertions, *CFB-MYH11*, *AML1-ETO* and *PML-RARA* in AML [158]; and *TP53*, *ATM*, *NOTCH1*, *BIRC3*, and *SF3B1* in CLL [159,160]. The amplification of Ig/TR can detect 10^−4^–10^−5^ but is labor intensive as each sample must be sequenced to identify patient-specific Ig/TR DNA sequences to design PCR primers that are used in the longitudinal testing. The challenge is that cancer cells undergo clonal evolution, hence gene rearrangements can change [142,161]. Molecular analysis provides high specificity and sensitivity but its limitation is costly, time-consuming, and complex workflows [162].

Novel MRD detection using circulating diseased cells from PB can utilize microfluidic-based MRD devices. Cell sorting and isolation using microfluidic devices followed by immunophenotyping have been demonstrated to offer the advantages of rapid, high-throughput, low-cost testing [73,76,77,163]. Similarly to circulating tumor cells (CTCs) in epithelial malignancies, CLCs can be isolated from PB using similar types of microfluidic devices [69,71,75,76,164,165,166,167,168]. MRD testing using PB offers important advantages in terms of the ability to provide more frequent testing as opposed to using a BMA sample. CLCs can be isolated from PB using a positive selection process in which affinity agents (i.e., antibodies, aptamers, and peptides [72,76,169]) attached to the surface of a solid support can be used to capture rare cells of interest that express specific antigens (i.e., CD19, CD34, CD117, CD33, and CD138) [73,75,76]. Due to the heterogeneity of some blood cancers, the monitoring of MRD requires multiple antigens to identify cancer cell subpopulations (e.g., AML uses CD34, CD117, CD33 and patient-specific aberrant markers) [74]. Affinity isolation of rare cells from blood has been widely used as it provides better specificity than physical methods [163].

CLCs can be separated from other blood cells based on differences in physical properties, such as size, shape, density, deformability, and dielectric constant without the need for labeling or tagging steps [170,171]. Microfluidic cell sorting and separation devices include those that use passive forces such as filtration, inertial focusing, deterministic lateral displacement (DLD), or active transduction such as dielectrophoresis (DEP), magnetic, and acoustic forces [170,171]. Cells with different phenotypical features but similar physical properties can be enriched using immunocapture via affinity agents [163,172].

Several critical reviews of microfluidic technologies including material characteristics were published for the isolation of rare cells including CTCs and CLCs from peripheral blood [173,174,175], and the molecular analysis of liquid biopsy markers [176,177,178]. A quantitative comparison of CTC isolation methods focuses on recovery, purity, throughput, viability, and marker bias [179]. Affinity-based cell isolation methods for blood cancers provide low biological specificity as both normal and cancer cells can express common antigens. If not designed properly, affinity selection may neglect orthogonal phenotypes of cancer cells; however, this can be addressed by using a mixture of affinity agents to select a wider range of antigens. Recovery in affinity-based methods is dependent on binding kinetics and thermodynamics, which in turn depend on antibody parameters and the expression level of antigens resident on the target cell, respectively. In contrast, size- and deformability-based microfluidic devices are phenotype-independent, making them potentially better suited for the isolation of heterogeneous populations of rare cells, but at a cost of purity and the need for single-cell picking prior to downstream analysis. Furthermore, while many technologies have been demonstrated for the isolation of CTCs, which have a larger physical size than blood cells, they are not ideal for the selection of CLCs owing to their similarity in size between blood cancer cells and normal cells.

Cai et al. [180] designed a microfluidic device, which integrated the principle of filtration and hydrodynamic methods for MRD monitoring in acute leukemia via CLC enumeration (Figure 3B). MRD is defined as the presence of the number of cancer cells that remain in a patient’s body after treatment. This device enabled highly efficient cell isolation at a wide range of flow rates without diluting the blood sample that has a large impact on device throughput; however, as the size distribution of leukemia cells overlaps with mature blood cells, a two-step filtration process with different cut-off sizes was needed to select cells with 16–18 µm diameters. This size-based cell enrichment device increased the concentration of target cells by two orders of magnitude while removing other blood cells at 4–5 orders of magnitude using focus-separation structures in series achieving a theoretical sensitivity of 1:10^−5^ in combination with morphological detection. Purity or sample loss for the isolated cells was not reported, which is a concern as size is the only criteria used for selection.

Microfluidic devices combining physical with affinity isolation were developed to improve interactions between target cells and the affinity agent. Liu et al. [169] reported a biomimetic Multivalent Aptamer Nanoclimber (MANC)-functionalized microfluidic chip (MANC-Chip) for minimally invasive, highly sensitive and clinically applicable MRD detection in T-ALL patients (Figure 3C). In this study, the MANCs have flexible structures and cooperative multivalent effects, which enhanced the affinity against T-ALL cells. The collision probability between MANCs and leukemia cells was further increased by a DLD structure design. High capture and release efficiency (92% and 89%, respectively) were achieved with 94% viability of the captured cells.

**Figure 4:**
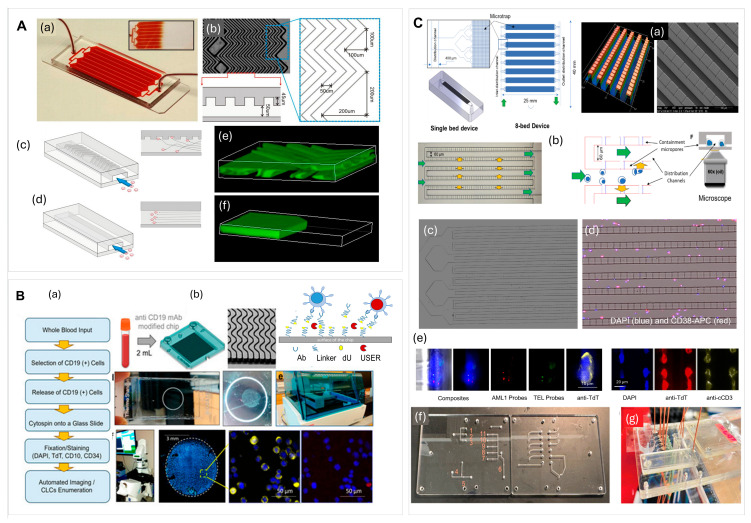
(**A**) The rendering, design, and flow pattern of a herringbone device used for cell isolation. (**a**) The herringbone device and inset illustrates the uniform blood flow through the device with laminar flow; (**b**) a micrograph of the grooved surface, illustrating the asymmetry and periodicity of the herringbone grooves; (**c**) a cartoon of the herringbone device; (**d**) a cartoon of a device without herringbone grooves; (**e**) a flow visualization showing chaotic microvortices generated by the herringbone grooves; (**f**) a flow visualization showing the lack of microvortices in the flat-walled device [71], reproduced from Stott et al. (2010). Copyrights National Academy of Sciences, 2010. (**B**) The schematic and workflow of a sinusoidal channel device utilizing CD19 affinity surface selection, release, and processing of CLCs from B-ALL PB samples. (**a**) A flow chart showing the entire workflow for processing patient PB samples to search for CLCs; (**b**) an illustration of PB processed using a microfluidic device modified with mAbs specific for CD19 with downstream CLC immunophenotyping and enumeration [76], reproduced from Witek et al. (2024). Creative Commons CC BY license. (**C**) The microfluidic network of a microtrap chip (a single bed and the corresponding 8-bed device are shown). Microtrap size: 4 µm × 2 µm × 50 µm (w × d × l). (**a**) A SEM image of a microtrap replicated in PDMS, and the deeper interleaving channels are also shown in the accompanying SEM image. (**b**, **left**) A schematic showing the operation of the microtrap chip. Cells in solution (green arrows) are contained at the entrances of the microtraps, letting the fluid pass (yellow arrows) into the outlet channels of the interleaving network; and (**b**, **right**) a schematic showing the 3-D model of cells captured in the microtrap chip and imaging objective through a thin cover plate [183]. (**c**) A brightfield image of the bifurcated entrance channels of the microtrap device. (**d**) A brightfield image merged with DAPI (blue) and CD38-APC (red) fluorescence channels showing an MM cell line (RPMI-8226) contained within the microtrap chip. Cells stained with DAPI (nuclear stain) and CD38 markers aligned at the microtrap entrances [183] (reproduced from M. Weerakoon-Ratnayake et al. (2020), Creative Commons CC BY license). (**e**) The fluorescence in situ hybridization (FISH) with CLC isolated from B-ALL patient and fluorescence immunophenotyping (DAPI, TdT, and cCD3) of MOLT-3 cell line (adapted from Ref. [112], CC BY). (**f**) A fluidic motherboard with channels and connections for modules and valves used in an integrated and modular microfluidic system for MRD detection with (**g**) 3 modules: cell selection module, jumper module, and imaging module [112] (adapted from Childers et al. (2024), Creative Commons Attribution 3.0 Unported License).

Khoo et al. [181] demonstrated a one-step CLC platform using a spiral microfluidic chip using inertial focusing, which is a high-throughput, label-free technique that uses fluid inertia to precisely manipulate cells based on size (Figure 3D); however, similar to filtration owing to CLCs’ and normal blood cells’ similar size, the purity of the isolated CLCs was low. As normal cells were co-isolated, immunostaining was primarily used for the identification of CLCs. This chip was validated for MRD monitoring in ALL, myelodysplastic syndrome (MDS), AML, acute monocytic leukemia (AMoL), and acute myelomonocytic leukemia (AMMoL) patients and demonstrated a sensitivity of 10^−6^ with 56% detection efficiency of CLCs compared to MFC from BMAs. A major disadvantage of the device was the need for pre-processing steps to remove RBCs prior to CLC sorting. A microfluidic device with herringbone structures developed initially for the isolation of CTCs [167] (Figure 4A) was used for CLC isolations. The herringbone structures delivered PB mixing, hence, good cell recovery owing to more interactions between target cells and the surface bound antibody. Lai et al. [182] used a CD34-coated herringbone chip with disulfide linkers to capture and release leukemic stem cells for AML monitoring. Leukemia stem cells were identified by immunophenotyping using CD123 and CD38 staining and isolated CLCs could be released for single-cell RNA-seq to check the different transcriptional profiles of stem cells subtypes. One mL of PB was processed at a flow rate of 25 µL/min with an isolation efficiency of 78% and a release efficiency of 76%.

Teixeira et al. [168] combined micropillars and herringbone structures on the same microfluidic device and modified the device surface with CD34 to isolate and preconcentrate AML blasts. The use of micropillars and herringbone structures provides lateral and vertical mixing, thus improving the capture efficiency. Different combinations of microstructures were analyzed using AML cell lines, with the highest capture efficiency of 55% at 40 µL/min PB flow. The isolated cells from AML blood samples were identified using aberrant markers (CD7 and CD56) inside the microfluidic devices. The disadvantage of the method is the need for sample pre-processing requiring mononuclear cells as input to microfluidic chips.

Sinusoidal microfluidics made of thermoplastics initially developed for epithelial CTC affinity isolation [60,165] have been used for MRD detection in different blood cancers [74,75,76]. These devices consist of 50–150 high-channels that provide fast (1.5–4.5 mL/h) processing of PB without the need for blood fractionation or any other pretreatment. The antigen/antibody encounter rates are induced by centrifugal forces in sinusoidal channels [184]. Jackson et al. [74] successfully monitored MRD for AML by isolating CLCs from PB samples using the device shown in Figure 4B. AML patients recovering from stem cell transplant were longitudinally tested for the presence of CLCs expressing CD33, CD34, and CD117 [73,74]. The isolated CLCs were released from the chip via the enzymatic cleavage of oligonucleotide linkers used to connect antibodies to the chip surface [185]. The released cells were enumerated and characterized by immunophenotyping using a patient-specific aberrant lineage marker (i.e., CD7 or CD56). The patients’ impending disease recurrence was detected 30 days earlier than standard of care methods [74]. The same microfluidic device modified with anti-CD19 antibodies were used to affinity select B-lineage ALL CLCs in PB for MRD monitoring in pediatric patients (Figure 4B) [76]. Isolated CLCs were released, enumerated, and analyzed via immunophenotyping with leukemia cells being positive for nuclear terminal deoxynucleotidyl transferase (TdT), or cytogenic analysis via fluorescence in situ hybridization (FISH), and molecular profiling using mRNA/gDNA. In comparison to MFC, this microfluidic offered high-sensitivity MRD detection (one leukemia cell among 200,000 normal mononucleated cells).

**Figure 5:**
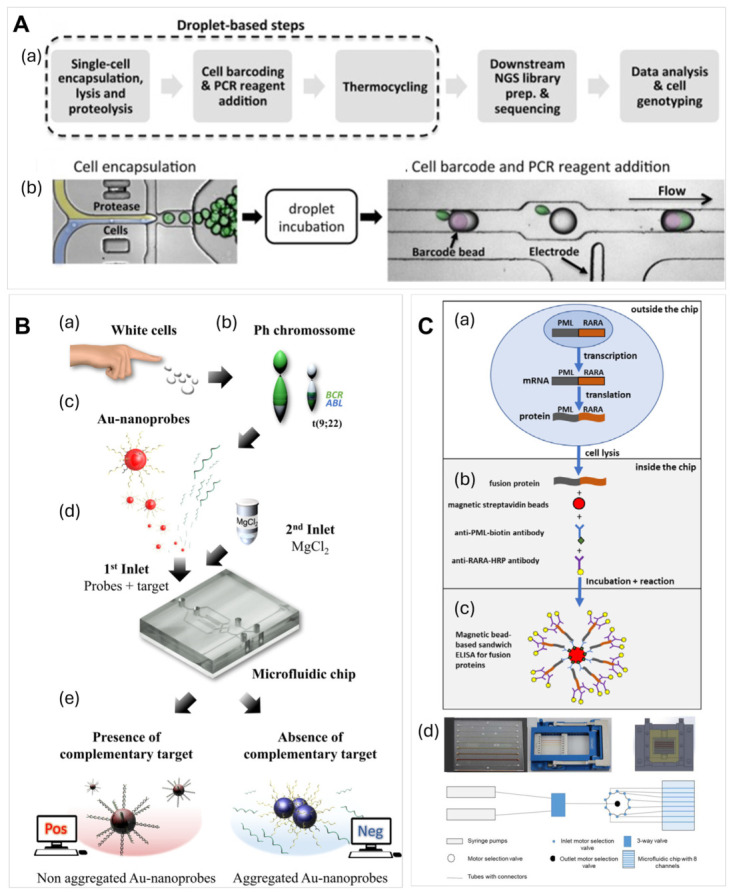
Microfluidic devices for the analysis of nucleic acids. (**A**) A droplet generation device used for single-cell encapsulation, genomic DNA amplification, and barcoding. (**a**) An overview of the workflow; (**b**) microfluidic devices to perform the two-step droplet workflow [84], reproduced from Pellegrino et al. (2018). Creative Commons CC BY 4.0 license. (**B**) A microfluidic device with colorimetric detection using gold nanoparticles functionalized with target sequence for RNA analysis. (**a**) WBCs extracted from a PB sample collected for analysis; (**b**) the presence of *BCR-ABL1* fusion transcripts, which is indicative of Philadelphia chromosomal aberration; (**c**) total RNA extracted from the WBCs and mixed with Au-nanoprobes functionalized with *BCR-ARL1* complementary sequences for hybridization; (**d**) Au-nanoprobes hybridized with RNA samples and salt solutions infused within a microfluidic chip; (**e**) the detection of *BCR-ARL1* based on color changes caused by the aggregation of the non-hybridized Au-nanoprobes [188], reproduced from Alves et al. (2018). Creative Commons CC BY 4.0 license. (**C**) A microfluidic for magnetic bead-based ELISA and showing the detection of PML-RARα. (**a**,**b**) The cell lysate is incubated with magnetic streptavidin beads, anti-PML-biotin antibodies, and anti-RARα, and horseradish peroxidase (HRP) antibodies; (**c**) anti-PML-biotin antibodies bind to the magnetic streptavidin beads. The anti-PML antibody binds to the PML region of the fusion protein while the anti-RARα-HRP antibody binds to the RARα region, (**d**) Images of the microfluidic chip consisting of 8 channels, chip holder, slider, and a schematic representation of the liquid handling system [85], reproduced from Emde et al. (2020). Creative Commons CC BY license.

Pellegrino et al. [84] reported a single-cell DNA analysis via two-step droplet microfluidic workflow (Figure 5A). Individual cells were encapsulated in droplets, lysed and merged with droplets containing reagents for amplification and molecular barcode-carrying hydrogel beads, which were sent to NGS [84]. This method was validated by analyzing longitudinally collected AML BMA samples, and loci that were implicated in AML progression were sequenced. The identified molecular features of clonal cells would not be discovered if bulk sequencing was performed. This method was later commercialized as the Tapestri single-cell DNA sequencing and has been used to study the mechanisms of juvenile myelomonocytic leukemia progression and evaluate the response of leukemia to therapy [186]. High-throughput gene expression analysis for ALL samples were performed using RNA extracted from CLCs via a microarray with TaqMan microfluidic cards [187]. The real-time PCR system used microfluidic cards with 384 microwells preloaded with TaqMan probes and primers, enabling a high-throughput and efficient real-time PCR-based assay.

Rapid RNA analysis without the need for amplification was developed using gold nanoprobes to perform RNA optical detection inside a microfluidic channel and used for the rapid detection of CML [188]. The microfluidic device (Figure 5B) combined a micromixer and an optical circuit to perform colorimetric detection. Total RNA extracted from WBCs was hybridized with gold nanoprobes functionalized with complementary sequences of the target gene. Color changes caused by the differential aggregation of oligonucleotide-functionalized Au nanoparticles were a detection transducer.

A droplet-based microfluidic with surface-enhanced Raman scattering (SERS) was demonstrated and tested using different leukemia cell lysates [189]. The lysates were mixed with SERS-active silver nanoparticles and enclosed in “water in oil” droplets. The SERS readout was performed in a quartz chip allowing for a high number of spectra to be collected, and based on the SERS signatures, leukemia cell lines were identified.

Immunoassays on microfluidic chips were demonstrated for blood disorders, specifically, acute promyelocyte leukemia (APL). Detection of an APL-specific protein called retinoic acid receptor α was performed using a microfluidic sandwich enzyme-linked immunosorbent assay (ELISA), Figure 5C [85]. The isolated antibody was bound to magnetic beads and the retinoic acid receptor α was detected with an antibody conjugated with horseradish peroxidase coupled with fluorescent readout of the signal. The ELISA was performed in a microfluidic channel allowing for continuous liquid processing and washing and, owing to short diffusional distances, the ELISA incubation times were short; the test was completed within 1 h.

### 2.4. Microfluidics for Lymphoma and Multiple Myeloma (MM) Diagnosis

Lymphoma is a cancer of the lymphatic system and develops from abnormal and uncontrollably multiplied lymphocytes, including B, T and natural killer (NK) cells [153]. Lymphoma can be classified into HL and NHL; the latter includes a variety of lymphomas such as diffuse large B-cell lymphoma (DLBCL), follicular lymphoma, and marginal zone lymphoma [190]. Similar to leukemia, lymphoma is diagnosed by MFC and molecular testing with gene rearrangements or chromosomal translocations after a biopsy of the lymph nodes [190,191,192]. Microfluidics were reported to aid in the diagnosis of lymphoma by the isolation of cancer cells and analysis of DNA/RNA/proteins [87,90,193,194]. Turetsky et al. [195] reported a microfluidic system for lymphoma cell capture and molecular analysis using sub-nanoliter traps, on-chip staining, and imaging. The system was tested using model cell lines for the central nervous system (CNS) lymphomas and artificial cerebrospinal fluid (aCSF) samples. Cells were isolated based on size and identified by B-cell markers CD19 and/or CD20, and kappa or lambda light chains (Ig clone identification). This device showed promise in CNS lymphoma diagnosis, subtyping, and therapeutic monitoring with fast, high-sensitivity results.

Yang et al. [196] reported an integrated microfluidic device for blood cell separation based on size and deformability and leukocyte identification based on morphology and immunophenotyping. With the aid of software for the image analysis of morphological cell features and immunophenotyping of CD3 or CD19 markers, an algorithm identified lymphoma cells from PB samples. The device enabled a single streamlined process offering a rapid method for detecting and subtyping lymphoma cells.

MM is a blood cancer (also called plasma cell disorder) with clonal proliferation of malignant plasma cells in the BM or circulating plasma cells (CPC) in PB, that excessively produce monoclonal Ig or light chains (i.e., M protein) [153]. Monoclonal gammopathy of undetermined significance (MGUS) and smoldering multiple myeloma (SMM), are clonal plasma cell disorders that can progress to symptomatic MM [197]. Diagnosis of MM requires blood and urine tests, whole body imaging, and BM examinations. Upon diagnosis, depending on type of disorder, standard treatments include chemotherapy, autologous stem cell transplantation, and immunotherapy [197,198]. Monitoring the efficacy of these treatments is important.

The isolation of CPCs from PB has been developed on diverse types of microfluidic devices. Most often affinity isolation methods target CD138-expressing CPCs; however, other targets such as B-cell maturation antigen (BCMA) appear to be of interest [77,199]. Qasaimeh et al. [200] reported a CPC microfluidic isolation device with herringbone mixer structures modified with an anti-CD138 antibody. The device reported the detection of <10 CPCs/mL and a capture efficiency of 40–70%. In situ immunophenotyping of isolated cells with anti-CD138 and anti-kappa staining was performed for CPC identification. Using the same type of device, but with a disulfide linker to attach the anti-CD138 antibodies to the capture surface for the nondestructive release of the isolated cells via reduction in S-S [201]. Kamande et al. [75] used a flow-through sinusoidal channel microfluidic chip fabricated in a thermoplastic for MM affinity capture with subsequent release of the selected CPCs [75,183]. CPCs were affinity isolated from PB with anti-CD138 antibodies covalently attached to the channel surfaces and enumerated via immunophenotyping with a mixture of fluorescently labeled antibodies to identify CPCs that were CD38(+)/CD56(±)/CD45(−). The isolated CPCs’ DNA was tested for the presence of *KRAS* mutations by PCR/ligase detection reaction (LDR) and chromosomal abnormalities by FISH [75]. The burden of CPC detected in PB correlated with disorder type; MGUS and active MM patients had the lowest and the highest burden of CPCs in PB, respectively [75]. This assay was further advanced to allow immunophenotyping and FISH to be performed on a microfluidic device [183] that was part of an integrated system for automated sample processing (Figure 4C) [59,112,183].

Ouyang et al. [202] reported a filtration-based micropillar device for CPC isolation and on-chip immunophenotyping to identify plasma cells as CD138(+), CD45(−), CD19(−). CPCs were filtered from PB based on size and stiffness with efficiencies of 40–55%, with the plasma cells recovered for further analysis by reversing the direction of solution flow. As expected, the selectivity of the method was low, typical for filtration-based cell selection.

Zeng et al. [203] combined negative affinity cancer cell isolation from MM patients’ BMAs, based on the physical properties of plasma cells. In this study, normal cells were first depleted by mixing and incubation with tetrameric anti-CD45 leukocytes-specific antibodies and removed by density centrifugation. The remaining WBCs were then infused into a DLD microfluidic chip for plasma cell enrichment based on size. The isolated MM CPCs were analyzed using flow cytometry and FISH to compare with the direct analysis of BMA samples. The new methodology showed improved sensitivity for cytogenetic abnormality detection, diagnosis, and risk stratification for MM. However, testing BMAs instead of PB was found to be a disadvantage.

A single-cell protein analysis digital microfluidic called “DMF-Protein-Seq” has been used for the identification and evaluation of protein expression in cells [204,205]. To quantify cell proteins, DNA-tagged antibodies with magnetic beads were used to affinity-label single cells. The DNA’s unique sequences served as barcodes for amplification and protein quantification and the magnetic beads allowed for magnetic-field-assisted cell manipulation [205]. Gao et al. [206] reported a microfluidic flow cytometer (FC) for the quantification of protein molecules on intact non-epithelial cancer cells of myeloid and lymphocytic lineages. The microfluidic FC was fabricated in quartz with the detection zone determined by the metal mask and channel dimensions (20 μm × 20 μm (w × h)). The uniform optical field for excitation and emission allowed for the quantification of proteins unevenly distributed on the cell. Three PMTs in series were sampling the fluorescence of traveling cells [206] followed by signal demodulation and processing. Dye-labeled antibodies were used to calibrate the system for antigen quantification. A microfluidic FC with a charge-coupled device (CCD) sensor operating in time-delayed-integration mode was reported by Hu et al. [207] for rare cell immunophenotyping. Leukemic B-ALL model cell lines were used to demonstrate the technology. The system employed a 488 nm laser and a spectrograph dispersing fluorescence along the CCD pixel columns to provide the immune-stained cell’s emission spectrum confined to rows of the CCD. The collected spectrum shifted in the serial register at a rate matching the B-ALL cell’s linear velocity. In such a configuration, the CCD’s shutter was always open, overlapping exposure with the readout of the previous frames providing a duty cycle approaching 100% [207].

### 2.5. Microfluidics for Non-Cell-Based Assays for Blood Disorders

Liquid biopsies (i.e., blood, plasma, urine, and saliva) provide access to not only cells but also other circulating markers such as exosomes or extracellular vesicles (EVs), cell-free circulating tumor DNA (ctDNA/cfDNA), circulating tumor RNA (ctRNA), proteins, immune markers, and metabolites [208]. Some of these liquid biopsy markers are of great interest for leukemia treatment efficacy because of their ability to provide molecular information on the disease. Wang et al. [209] has reported a microfluidic digital PCR system, which consisted of a micropatterned super-porous absorbent array chip (μSAAC), and tested its capability for detecting mutations in lymphoma. The μSAAC used PDMS microwells packed with dry agarose beads that absorbed the PCR mix with DNA template to form nanoliter compartments within the microbead. The device had the advantages of low-cost, spontaneous filling for the compartmentalization of target molecules without the need for a fluidic system, and the ability to prepare samples for sequencing.

cfDNA utility is now emerging as a biomarker in hematological diseases as well [210,211]. For example, cfDNA was evaluated via an allele-specific RT-qPCR with patient-specific gene rearrangements of pediatric B-ALL patients during induction therapy [212]. Plasma cfDNA abundance increased during the first days of treatment due to tumor cell death and after 4 days of treatment decreased owing to clearance from PB. The assay demonstrated its ability to track cfDNA kinetics and its concordance with the test performed on WBCs’ gDNA but with no advantage in analytical sensitivity [212]. Others have reported clinical indications based on cfDNA IgH rearrangements in patients diagnosed with aggressive NHL [213]. Heestermans et al. [214] used cfDNA’s methylation patterns and, distinct from healthy donors’ gDNA and cfDNA, epigenetic characteristics in MM. When methylation patterns of cfDNA and CPCs’ gDNA were compared to BM-derived gDNA, cfDNA showed the highest concordance of differentially methylated regions in BM [215].

Microfluidics technologies have also been used for circulating DNA and RNA analysis. Solid phase extraction methods used for DNA/RNA/protein pre-concentration and enrichment have used magnetic beads [216], or have been designed with fluidic architectures [83,217,218,219] with functionalized surfaces to improve the recovery of the circulating targets from the clinical sample to perform the subsequent molecular analysis [220,221].

EVs, including exosomes, microvesicles, and apoptotic bodies, are small vesicles released by cells through different biogenesis pathways [222]. EVs carry molecular cargo (i.e., proteins, lipids, and nucleic acids) associated with the cell-of-origin and thus have the potential to serve as biomarkers for disease screening, prognostication or diagnosis [223,224]. These biomarkers are isolated using methods such as ultracentrifugation, size-exclusion chromatography, and precipitation [225,226]. More recently, microfluidics have been used for the enrichment of EVs using for example immuno-affinity methods [80,227,228,229] and different architecture microfluidic architectures [79,230] using microfiltration [78], inertia [101,231], DLD [231], viscoelastic forces [232], flow fractionation, acoustic, and electrokinetic forces [80,227]. In fact, integrated system for EV analysis including a chip for the immunocapture and EVs quantification using immuno-fluorescence, provided full process automation [233].

Platelet-derived EVs in SCD patients were shown to activate neutrophils and vascular cells to form platelet–neutrophil aggregates that occluded pulmonary arterioles and contributed to acute chest syndrome, one of the leading cause of mortality in patients diagnosed with SCD [234]. Studies have also shown that plasma-derived EVs can be used as potential biomarkers for MRD monitoring of AML [235,236], MM [237,238], and lymphoma [239]. However, challenges exist in the isolation and enrichment of EVs derived from leukemic plasma, with challenges associated with the downstream analysis of their proteins and RNA due to mass limitations. Microfluidics can dramatically improve the efficiency of isolation and provide high specificity compared to benchtop methods.

## 3. Blood Disorders and Microfluidics

### 3.1. Anemia Diagnosis, Treatment, and Fundamental Studies

Anemia is one of the most common blood disorders worldwide, characterized by a reduction in RBC counts or a decrease in hemoglobin (Hb) levels, leading to impaired oxygen delivery to tissues [240]. Anemia is defined by Hb concentrations < 12 g/dL [1] resulting from either blood loss, nutritional deficiencies, destruction of RBCs, decrease in RBC production due to BM problems, chronic disease, or genetic abnormalities [241,242]. Traditional detection and diagnostic methods include complete blood counts (CBCs), PB smear examinations, and hematocrit (Hct) tests. These methods are well-established but suffer from low throughput, large sample volume requirements, expensive instrumentation, and the need for trained personnel [243,244].

Hb levels are a commonly used clinical indicator for anemia [1,241,242]. Hb is the protein complex in RBCs that is responsible for oxygen transport; a decrease in Hb levels will reduce oxygen levels in the blood, leading to dizziness, fatigue, shortness of breath, and abnormal heart rate [242,245]. Conventional methods for Hb measurements include automated hematology analyzers, hemoglobinometers and colorimetric assays. These methods’ limitations such as complex analysis using expensive instrumentation [246] make it difficult to meet the needs for POCT, especially in resource-limited settings. Microfluidics, on the other hand, can fill this gap by offering integrated, low-cost, miniaturized and simple methods capable of rapid and accurate Hb analysis.

Microfluidic methods have been developed for anemia diagnosis owing to their advantages of low-cost testing, minimal sample requirements, high sensitivity, POCT capability, and the ability to integrate with advanced detection techniques [99,244,247]. Microfluidic devices for Hb testing were developed with a readout based on absorbance [248], osmotic hemolysis, image processing [249], and colorimetric assays [250]. Recent demonstrations of microfluidic applications in studies on anemia are shown in Table 3. These include evaluations of physical and rheological properties of RBCs [251,252,253], oxygen transport [254,255], RBC adhesion and interactions with blood vessels [256,257,258], and hemolysis and oxidative damage of RBCs under stress [259,260]. These microfluidic devices enable precise control of the cellular microenvironment and physiological simulation of microcirculation, providing insights into the mechanisms of biological processes related to anemia for use in drug screening and the development of therapeutic strategies.

**Figure 6:**
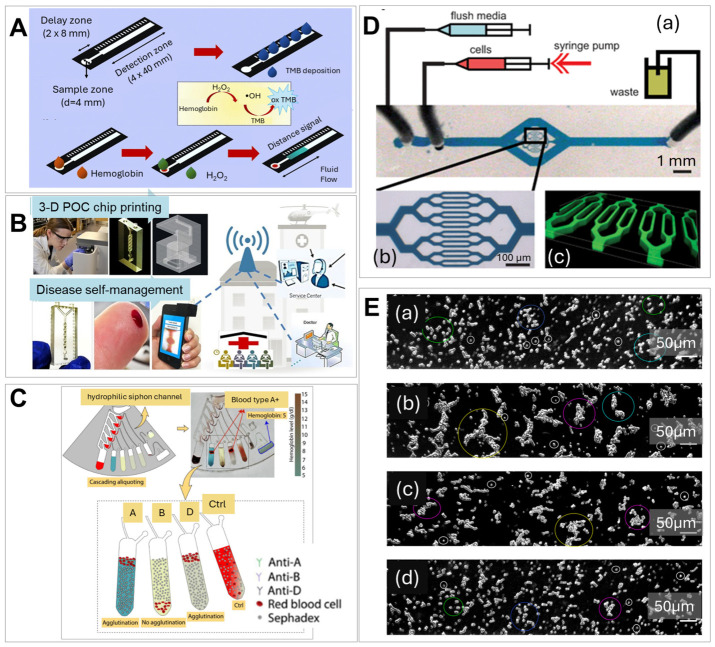
Microfluidic devices designed for RBC analyses. (**A**) Microfluidic devices for Hb measurements: a distance-based paper analytical device for Hb quantification [265], reproduced with permission from Khachornsakkul et al. (2025). Copyright by Royal Society of Chemistry, 2025. (**B**) A low-cost, smartphone-based 3D-printed microfluidic chip for Hb measurement and anemia diagnosis [113], reproduced with permission from Plevniak et al. (2016). Copyright AIP Publishing, 2016. (**C**) The schematic of a design and workflow of a centrifugal-based microfluidic device with colorimetric assay for blood typing and Hb measurements [266], reproduced from Dehghan et al. (2026). Creative Commons CC BY license. (**D**) A biophysical flow cytometer for cell mechanical property measurements to detect RBC deformability. (**a**) A schematic of the device; (**b**) a close-up image of the microchannels, (**c**) a confocal image of fluorescein solution inside several of the microchannels [267], reproduced with permission from Rosenbluth et al. (2008). Copyright Royal Society of Chemistry, 2008. (**E**) Images of RBC aggregation obtained with the BioFlux microfluidic system: (**a**) healthy individuals; (**b**) patients with CLL; (**c**) patients receiving Obinutuzumab/Venetoclax; and (**d**) patients receiving Ibrutinib. Color circles show RBC aggregates while white circles show unaggregated RBCs [268], reproduced from Alexandrova-Watanabe et al. (2025). Creative Commons CC BY license.

Khachornsakkul et al. [265] reported a distance-based paper microfluidic for rapid quantitative Hb detection in serum using 2 μL of sample within 10 min. Device design (Figure 6A) included sample and reagent fluid flow enabled by paper capillary action, which eliminated the need for external pumps. The so called “delay zone” between sample loading and the detection window allowed for optimized detection by regulating the fluid flow speed and reaction time of the catalytic colorimetric reaction to detect Hb. The concentration of Hb was measured by a naked eye reading the color on the paper strip, which eliminated the need for external reading instruments. The device showed a LOD of 2 mg/dL and a linear range up to 10 mg/dL with high accuracy (RSD < 2%).

A microfluidic diagnostic strategy for the low-cost and fast detection of Hb levels in blood (Figure 6B) was demonstrated by Plevniak et al. [113] using a 3D-printed integrated microfluidic chip and a smartphone camera for data collection. Blood was mixed with oxidizing agents in an auto-mixer for efficient colorimetric Hb detection with a 1 s reaction time. A camera and image analysis application were successfully implemented for anemia monitoring for health self-management.

Dehghan et al. [266] demonstrated an integrated centrifugal microfluidic platform for POCT of blood typing and simultaneous Hb level detection. The system employs Sephadex G-10-treated siphon channels for blood transfer and low volume sample metering (Figure 6C). The system combined the principles of gel column agglutination for blood typing and a colorimetric assay for Hb measurement. Images collected by the low-cost, 12-megapixel compact camera with autofocus were analyzed with a custom image-processing algorithm to provide an automated readout of both agglutination patterns and colorimetric intensity for Hb measurement. This system enabled portable and rapid, low-volume (12 µL) Hb quantification for securing important information for transfusion-related testing in anemia patients in resource-limited areas.

Another important clinical indicator for anemia is RBC count via Hct, which measures the percentage of RBCs in whole blood. Traditional methods rely on blood centrifugation and hematology analyzers. The automated hematology analyzer price is ~$15k, with a per-test cost of ~$10 [52]. Owing to bulky instrumentation, the tests are done in clinical laboratory settings only.

Paper-based tests have been widely used for rapid POCT due to their simplicity and disposability, low cost, and ease of fabrication. Ram et al. [269] developed paper-based devices for simultaneous Hct and Hb measurements. The device measured Hct levels by analyzing the area of a blood drop spreading on filter paper. Hb was measured by the color intensity of lysed RBCs. The reported results were comparable to those secured from automated hematology analyzers. Another demonstration of Hct measurement was based on centrifugal microfluidics [270]. The controlled rotation of centrifugal microfluidics separated plasma and packed cells by density and electrical impedance spectroscopy was used for Hct enumeration. Compared to the cost of the paper-based method ($0.03 per device [271]), the centrifugal microfluidic tests ($3 per device [272]) were more expensive and, additionally, required dedicated instrumentation. Both assays, however, were more economical than a benchtop hematology laboratory test. Further validation tests are needed to compare these two microfluidics’ figures of merits.

A notable example of a successful POCT is the HemoTypeSC™ test [273] developed for SCD diagnostics based on the measurement of sickle hemoglobin (HbS). This competitive lateral flow immunoassay with monoclonal antibodies for the determination of the presence of different Hb genotypes offers the advantages of being a cost-effective (~$2/test), simple and fast test to diagnose SCD [274].

### 3.2. Blood Rheology and Mechanical Properties of Blood Cells

Hemorheological alterations are commonly observed in anemia and other diseases such as diabetes, hypertension, obesity, cerebrovascular and peripheral vascular diseases. Biophysical abnormalities of RBCs, including changes in cell size, shape, and membrane integrity, are linked to alterations in blood viscosity, RBC aggregation, and deformability, all of which affect microcirculation and microvascular perfusion [275,276]. Conventional methods for the study of RBC biophysical properties include bulk cell methods (membrane microfiltration and ektacytometry [277]), and single-cell imaging methods (i.e., AFM) and optical tweezers [278]. These methods are limited in the ability to study RBC mechanics and behavior under physiological microenvironment and flow conditions [279]. With its advantages in precise fluid flow control and the ability to probe single-cell behavior, microfluidics has emerged as a powerful tool for studying the micro-rheology of blood and the mechanical properties of blood cells with high precision and throughput.

Microfluidic imaging cytometers and constriction channels have been widely used to study RBC morphology, deformability, and aggregation, as well as leukocyte aggregation and adhesion, which serve as markers for hematological diseases such as anemia and SCD [275]. Various techniques have been developed to measure RBC deformability, with two dominant approaches: single-cell measurements of individual RBCs and bulk measurements of whole blood and RBC suspension [275]. Rosenbluth et al. [267] developed a high-throughput microfluidics-based PDMS “biophysical” flow cytometer (FC) (Figure 6D) that analyzed single-cell transit time, to get information on the cell size and deformability. Clinical applications of this technique were demonstrated in sepsis and leukostasis (i.e., high WBC counts), in which the mechanical properties of cells lead to microvascular obstruction. Similar cytometry-based devices were also used by Eluru et al. [280], Guruprasad et al. [281], and Wei et al. [282].

RBC aggregation is an important parameter governing hemorheological properties and is related to blood viscosity. It is typically measured using photometric intensity, electrical conductivity, or via microscopic observation [283]. With advances in image processing technology, the combination of image analysis and microfluidic devices is increasingly used to accurately evaluate cell aggregation. Alexandrova-Watanabe et al. [268] assessed the extent of RBC aggregation in CLL patients and the effects of therapeutic drugs by developing a new algorithm for software image flow analysis to model RBCs in microchannels simulating blood vessels. A commercially available air-pressure-driven microfluidic system (BioFlux) was used to study RBC aggregation, as well (Figure 6E). The image-based RBC aggregation analysis method provided detailed information about RBC aggregate size and types, elucidating the mechanisms of aggregation.

As blood viscosity can be related to many diseases or conditions that affect its proper flow properties, microfluidic chips were used for blood viscosity measurement. The movement of only 20 µL of blood was monitored to evaluate viscosity-related parameters based on the Hagen–Poiseuille equation [275,283]. For example, Kang et al. [284] have developed blood viscometers using micro-particle image velocimetry to measure blood velocity, [284,285] and time-lapse blood velocity [286]. A smartphone camera was also used as a detection tool, enabling a wide range of detection and analysis capabilities [114,287,288].

### 3.3. Hemophilia, Thrombosis and Platelet Disorders

Blood disorders related to bleeding and clotting include hemophilia, thrombosis, and platelet disorders that disrupt normal hemostasis. Hemophilia is an inherited bleeding disorder caused by deficiencies in coagulation factors VIII or IX [289]; thrombosis typically occurs in vascular regions with disrupted blood flow and is associated with endothelial damage, a hypercoagulable state, and hemodynamic alterations [290]. Platelet dysfunctions are related to genetic autoimmune diseases [291]. Typical diagnostic and screening methods for bleeding and clotting disorders include coagulation assays, such as prothrombin time and activated partial thromboplastin time [292], which are time-consuming and provide limited information. Microfluidics has been widely applied to study hemostasis and platelet function under physiologically relevant flow conditions, as well as for diagnosing bleeding disorders and drug screening [293,294].

**Figure 7:**
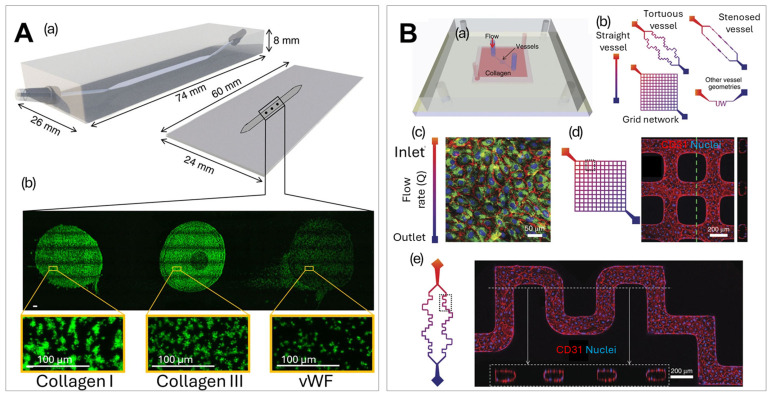
Microfluidic devices used for platelet aggregation and adhesion studies. (**A**) A rendering of single flow channel using micro-spot arrays of protein-specific platelet-adhesive surfaces and images of those micro-spots highlighting the differences in platelet adhesion. (**a**) A schematic of the parallel-plate flow chamber; (**b**) florescence images showing the adhesion of platelets to micro-spots with different surface coatings [295], reproduced from De Witt et al. (2014). Creative Commons CC BY 4.0 license. (**B**) Micro-vessel platform designed to replicate vascular environments for von Willebrand factor (vWF) fiber formation monitoring. (**a**) A schematic of the micro-vessel system in collagen gels and (**b**) different micro-vessel geometries. The images in (**c**–**e**) show endothelial cells seeded and cultured in channels with different geometries to achieve a continuous endothelium—red: CD31; green: VWF; and blue: nuclei, dotted lines show z-projection of confocal sections [299], reproduced from Zheng et al. (2015). Creative Commons CC BY 4.0 license.

Platelet aggregation and adhesion are essential processes during hemostasis and thrombosis. Multi-micro-spot-based microfluidic arrays (Figure 7A) were developed to study different platelet adhesive proteins in thrombus formation and to help identify patients with bleeding disorders (i.e., hemophilia) [295,296,297]. Blood samples were perfused through flow chambers containing micro-spots modified with different platelet adhesive receptors to form thrombi. Thrombi characteristics were evaluated via staining and imaging to assess platelet function and correlate with disorders.

Microfluidic devices with engineered three-dimensional channels (i.e., micro-vessels) consisting of perfusable collagen lined with endothelial cells (Figure 7B) [298,299] were used to study the mechanisms of hemostasis and thrombosis, thereby advancing the understanding and treatment of thrombosis [300,301]. The endothelial-like system of in vitro micro-vessels was engineered by seeding human umbilical vein endothelial cells into microfluidic structures formed in a type I collagen gel. The endothelial cells self-organized and formed microvascular tubes and released von Willebrand factor (vWF). In the study by Rayner et al., the interaction between vWF and fibrinogen were examined using a 3D micro-vessel system [300]. Binding characteristics or lack thereof between fibrinogen or fibrin monomers with vWF were measured at the single molecule level by the colocalization of fluorophore-labeled recombinant proteins within a microfluidic flow chamber. The micro-vessel system provided a physiologically relevant environment that mimics the in vivo vascular system by precise control of fluid flow, shear stress, and cellular composition. The design enabled real-time monitoring of clot formation and provided insights into the fundamental mechanisms of thrombotic diseases.

Establishing this disease model on a microfluidic platform requires adequate time and carefully controlled conditions for platform preparation. Typically, approximately five days are needed to culture endothelial cells to confluency within the microchannels, followed by system validation before thrombosis formation can be assessed by microscopy. This stands in contrast to conventional coagulation assays: prothrombin time (PT) and activated partial thromboplastin time (aPTT) measurements can, in principle, yield results on the clotting process in under a minute [302].

However, this difference in throughput is offset by the richer mechanistic information that microfluidic models provide. Unlike PT and aPTT, which report a single endpoint, microfluidic vascular models offer direct insight into shear-stress-mediated and cell-mediated thrombogenesis, enable systematic evaluation of the contributions of individual factors to thrombus formation, and support the identification of novel therapeutic targets for thrombotic disease. Furthermore, microfluidic platforms can model clinically relevant causes of abnormal PT and aPTT results—including vitamin K deficiency, sepsis, and factor VII deficiency—allowing these conditions to be interrogated in a controlled, dynamic hemodynamic environment that static benchtop assays cannot replicate [302].

Microfluidic devices can replicate in vivo shear rate conditions, exposing biological cells to physiologically relevant levels of fluid shear stress. Shear stress is proportional to blood viscosity and inversely proportional to the cube of the channel, vein, or capillary radius. Consequently, microfluidic channel dimensions can be precisely designed to generate a range of shear stress values that match specific physiological environments. Owing to blood’s non-Newtonian characteristic, meaning its viscosity is not constant but varies with shear rate, the accurate modeling of hemodynamic conditions requires careful control of linear flow rate velocity. A key advantage of microfluidic systems is their ability to process undiluted whole blood, enabling precise replication of the desired shear conditions without the confounding effects of sample dilution. In humans, normal physiological blood flow produces a shear stress of 0.03–1 dyn/cm^2^ at the surface of epithelial and endothelial cell linings [303], 1–6 dyn/cm^2^ within the venous system, and 10–70 dyn/cm^2^ in the arterial vasculature [6,7]. Microfluidic devices have been demonstrated across this full physiological range: systems capable of processing blood at shear stresses of 14 dyn/cm^2^ have been reported [304], and platforms operating at higher shear stresses of up to 54 dyn/cm^2^ have also been developed for cell-based applications [183].

## 4. Microfluidics for Disease Modeling

### On-Chip 3D Cell Culture for Blood Disorders

3D culture can mimic and model the cell environment and are tools for drug testing and preclinical studies for several types of cancer. Mulholland, T. et al. [95] designed a multi-layered PDMS-based microfluidic platform with microwells for cancer drug screening, where cells were sedimented at the bottom of the microwells and aggregated into compact multicellular spheroids. The device was used for the co-culture of CML cells with stroma cells and mesenchymal stem cells, and investigated cell response to different treatments [305]. Two media reserves on either side of the device allowed for the creation of a concentration gradient which was used to test cells’ sensitivity to drugs. Spheroids were stained and imaged for the viability and colocalization of cells.

**Figure 8:**
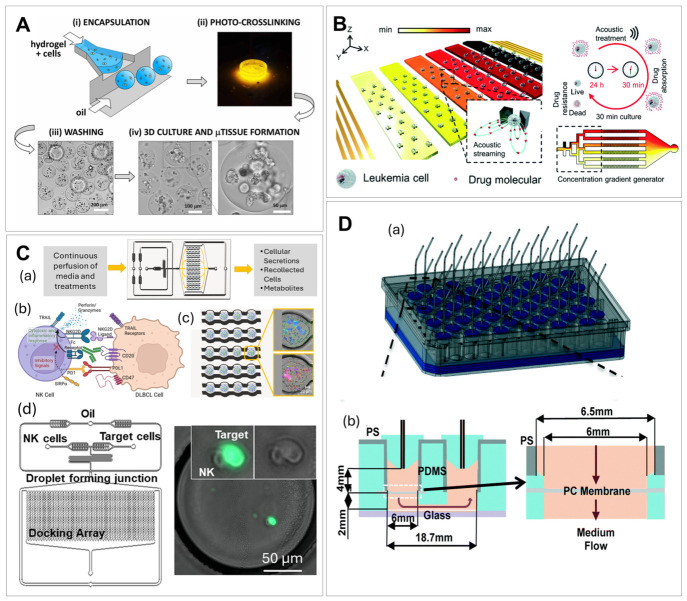
(**A**) Multi-cell encapsulation via droplet generation for 3D tissue modeling and drug testing. The workflow of cell encapsulation using a droplet microfluidic [306], reproduced from Rudzinska-Radecka et al. (2025). Creative Commons CC BY 4.0 license. (**B**) A single-cell drug screening device with cell trapping channels containing different drug concentrations and acoustic streaming to accelerate drug uptake. The design of the microfluidic device and experimental process for rapid drug screening in a drug gradient microfluidic chip based on high-frequency acoustic-wave-induced acoustic streaming [98], reproduced with permission from Zhao et al. (2021). Copyrights Royal Society of Chemistry, 2021. (**C**) Droplet generation used to co-culture lymphoma cells and NK cells with a built-in docking array for tissue modeling and drug testing. (**a**) A drawing of the 3D device filled with cell-loaded hydrogel spheroids; (**b**) a diagram depicting some of the biological characteristics observed in NK cells and DBLCL cultured using the spheroid platform; (**c**) images of spheroids containing DLBCL and NK cells before and after rCHOP treatment [90], reproduced from Sullivan et al. (2024). Creative Commons CC BY 4.0 license. (**d**) NK cell dynamics in a droplet microfluidic device; images showing contact between NK (unlabeled) and SUDHL10 (Calcein AM labeled) cells in droplet [193], reproduced from Sarkar et al. (2017). Creative Commons CC BY license. (**D**) Microwell plate-based perfusion culture device for cell culturing within a tumor microenvironment. (**a**) Schematic illustrations of the prototype device assembled on a commercial polystyrene bottomless 96-well plate. (**b**) The structure of fluidic channels between the inlet and outlet chambers and PC membrane disks in culture wells [307], reproduced with permission from Zhang et al. (2015). Copyrights Royal Society of Chemistry, 2015.

Droplet-based microfluidic spheroid models have also been demonstrated to assess treatment conditions in parallel. Droplets encapsulated myeloid leukemia cells and cells from BM to build a 3D model of the leukemic BM microenvironment and leukemia–stroma crosstalk in CML, Figure 8A [306]. The high-throughput spheroid production method using the microfluidic provided low reagent consumption and excellent reproducibility and would potentially make this drug screening platform useful for the rapid screening of anti-leukemia therapeutics. A chip for drug screening was designed with physical cell traps allowing cell manipulation, which was used as a non-invasive single-cell rapid drug screening method for leukemia cells, Figure 8B [98]. This system combined high-frequency acoustic waves with a concentration gradient microfluidic chip, in which the high-frequency acoustic waves induced shear force that increased cell membrane permeability, significantly improving the drug uptake process. This chip provided a way to overcome the diffusion-limited uptake of drugs at the single-cell level and while is promising for future drug screening owing to high throughput, low reagent input and rapid response, the chip did not reproduce true drug uptake by cells.

A 3D culture model of cancer and the vascular microenvironment was used to study therapy for lymphoma under physiologically relevant conditions. The microfluidic droplet generation system monitored NK cell interactions with B-type NHL cells at the single-cell level by encapsulating both NK and lymphoma cells together in picoliter-volume droplets, Figure 8C [193]. The assay identified significant heterogeneity in distinct types of cell interactions and offered an analytical tool for assessing anti-NHL immune responses and the evaluation of NK-based immunotherapies.

A high-throughput immunogenic DLBCL spheroid generation system was reported by Sabhachandani et al. [308], which consisted of lymphoma cells, fibroblasts, and lymphocytes within an alginate-PuraMatrix™ hydrogel. This model enabled the dynamic analysis of cellular immune interactions, proliferation, and cytokine secretion. The system was tested for immunomodulatory drug responses and showed potential for high-throughput anti-cancer therapeutic screening (Figure 8C) [90], where primary patient DLBCL cells and NK cells were co-cultured and tested for their response to chemotherapy. Cellular viability in response to treatment, rheological properties, and cell surface marker expression levels correlated with the in vivo characteristics.

Lamaison et al. [309] reported a microfluidic encapsulation method to incorporate lymphoma B cells, extracellular matrix (ECM), and/or tonsil stromal cells (TSCs) inside permeable, elastic, and hollow alginate microspheres and used it as a tunable 3D model for co-cultures of follicular lymphoma cells with ECM and TSCs. The 3D spheroid model was used to study interactions between lymphoma B cells and stromal cells as well as drug response to chemotherapy. This high-throughput 3D model mimicking the lymphoma tumor niche could be used for studying the dynamics of lymphoma B cells and the microenvironment as well as cancer drug screening.

Biomimetic vascular models were used to study microenvironment and treatment responses for lymphoma. Anastasiadou et al. [310] employed a 3D vascular model to assess T-cell response to EBNA2-transfected DLBCL cells and the immunogenicity of the EBNA2-transfected DLBCL cells. Mastini et al. [311] used a 3D anaplastic lymphoma kinase (ALK)-driven anaplastic large cell lymphoma (ALCL)-vascular microfluidic chip for the co-culture of endothelial cells and ALCL cells and to evaluate their response to ALK tyrosine kinase inhibitors with a tumor microenvironment. These studies showed the application of biomimetic vascular models in mechanistic studies as well as therapeutic response evaluation.

A well-plate-based cell culture system was developed by Zhang et al. [307] and applied for the culturing of primary patient-derived MM cells (Figure 8D). The platform consisted of a 96-well plate modified perfusion culture device, where the wells were connected via PDMS structures with a polycarbonate membrane to reconstruct the tumor microenvironment. The use of a standard well plate made it compatible with conventional benchtop techniques, such as well plate readers, PCR, and flow cytometry. This system was validated by the co-culture of patient BM mononucleate cells with ossified tissue pre-grown from osteoblasts (OSBs), and was analyzed for MM cell proliferation, the viability of OSB, and the drug response of OSBs and MM cells. This system was used by the same group to study interactions between MM cells and OSBs and co-culture conditions [91]. A similar design was used to visualize MM cell trafficking through the sinusoidal niche of the BM under physiologic conditions [93]. The well plate culture system was also modified to be a pumpless platform with the addition of a programmable rocking device to enable gravity-driven flow control during cell culture [312].

Ben-Arye et al. [96] reported a tissue-on-chip platform with stationary nanoliter droplet arrays integrated with individually addressable electrodes for each droplet (i.e., microwells for drug) and used lymphoma cell lines. Changes in crossover frequency for dielectrophoresis were monitored for each lymphoma cell treated with molecular inhibitors and cytotoxic drug. The system showed potential for a label-free, non-invasive drug screening tool for lymphoma management.

The BM microenvironment has been studied using microfluidic “tissue-on-a-chip” models to evaluate disease progression, cell interactions, and therapeutic responses for MM. The maintenance of patient-derived cell culture on a chip was explored for personalized drug screening and biomarker discovery [94,307]. The microfluidic cell culture and modeling system enabled the precise control of nutrients and drug flow along with integration for cellular and molecular analysis. A hanging droplet technique for 3D tissue culture has been combined with an open well designed microfluidic and applied for long-term culture to evaluate different treatments on MM model cells [313]. The microfluidic droplet array system for the dynamic analysis of interactions between dendritic and T cells and MM cell lines at the single-cell level can be applied for the future evaluation of cell-based immunotherapies [314]. The device was used to study a variety of other cell interactions [315]. Carreras et al. [316] presented a microfluidic 3D culture device using hydrogel droplets to recapitulate the niche of patient-derived MM cells. The double layered hydrogel beads allowed successful culture of MM cells with and without supporting mesenchymal stem cells.

Microfluidic devices offer the possibility of actively controlling and transporting analytes, cells, or particles through applied forces that enable precise micro-manipulation [317]. Microfluidics can also be configured as passive systems without active hydrodynamic flow making them well suited for modeling chemotaxis experiments in which hydrodynamic forces would otherwise confound the biological signal [318]. A key challenge in such experiments is distinguishing active cell migration, induced by a chemo- or bio-attractant, from that of passive displacement resulting from Brownian motion or fluid-driven transport. Resolving these differences is essential for the correct interpretation of cell behavior. Microfluidic devices that incorporate membranes or hydrogel barriers restrict bulk fluid flow while allowing the diffusion of molecules and concentration gradients without subjecting cells to hydrodynamic forces. This design strategy eliminates flow-driven cell displacement, ensuring that any observed cell movement is attributable to active, gradient-directed migration [319].

## 5. Microfluidics for Chimeric Antigen Receptor T-Cell (CAR-T) Manufacturing

CAR-T therapies have been developed and approved by the FDA for lymphoma and ALL in the last decade [320]. In CAR-T therapy, genetically modified T cells with CARs designed to recognize specific tumor antigens are infused into the patient’s blood to attack cancer cells [321,322]. Owing to the attractive features of microfluidics such as a miniaturized reaction system, precise control of fluid flow, and the ability to manipulate target cells at the single-cell level, microfluidics has been applied for various aspects of the CAR-T therapy workflow. As a first step of CAR-T therapy, the isolation of T cells from a patient without contamination of tumor cells is essential. If tumor cells are genetically modified along with T cells to produce CAR molecules this will make them immune to CAR-T cells [323].

Different microfluidic platforms have been developed to purify primary T cells before gene transfer, including label-free methods and affinity isolations [323]. Microfluidic devices based on DEP forces were used to separate tumor cells from regular CD8+ T cells, as tumor cells typically produce large electric polarizability compared to normal T cells [323]. Both CML and B-ALL cell lines were evaluated and 100% purity with high cell viability >90% were achieved. Another label-free T cell purification method using inertial microfluidics was reported by Elsemary et al. [324]. The same type of device was used to purify CAR-T cell products by removing dead cells and debris while maintaining the CAR-T cell function, proliferation potential, and cytotoxic actions [325]. Jeon et al. [326] separated inactive from active T cells before CAR engineering, improving CAR-T cell manufacturing efficiency. Affinity isolation and separation of leukemic B cells from T cell populations using CD19-labeled magnetic nanoparticles and a microfluidic device attached to permanent magnets was reported [327].

Liu et al. [328] introduced a high-throughput microfluidic system to synthesize antigen-presenting cells (APCs) that mimic the viscoelastic T-cell activation of natural APCs. The “synthetic” cells enhanced the expansion of CD8(+) T cells and CAR transduction efficiency to ~90% for B-cell lymphoma. Sin et al. [194] developed an integrated microfluidic bioreactor for the automated production of CD19 CAR-T cells for lymphoma treatment, including activation, transduction, and expansion all in one device. This device enabled continuous environmental monitoring and control of the CAR-T cell cultures. The device could be redesigned for high-throughput optimization and larger-scale CAR-T production.

An analysis of the CAR-T cells to evaluate their functions using a microfluidic platform can provide single-cell-level information of the CAR-T cells before infusion, help predict the efficacy and immunotoxicity of the therapy, and improve the CAR-T production process. Xue et al. [329] employed a single-cell, 16-plex cytokine microfluidic device, a so called single-cell barcode chip for the evaluation of CD19 CAR-T cells targeting B-cell blood cancers, such as B-ALL and DLBCL. Stimulated CAR-T cells were suspended onto a single-cell barcode microchamber array chip containing ~12,000 microchambers to distribute cells for imaging. A 16-plex antibody barcode slide was used to analyze CAR-T cytokine production at the single-cell level. This microfluidic device enabled comprehensive characterization of the CAR-T cells’ functions and revealed the multifunctional heterogeneity of CD19 CAR-T cells. A similar study evaluated the immunotherapy efficacy of CAR-T cells performed on a graphene oxide quantum dot (GOQD)-based microfluidic chip [330]. The antibody barcode slide employed the GOQD for antibody immobilization with the capacity to provide uniform, stable, low background detection of cytokine secretion by CAR-T cells. Other than cytokine secretion, commercially available microfluidics droplet generation devices were used to generate droplets containing a single CD19 CAR-T cell and target lymphoma cells to assess the cytotoxic effect from CAR-T cells [331].

Another field of microfluidic application for CAR-T therapy lies in the gene transduction and editing of the CAR-T. Unlike traditional viral or non-viral (e.g., liposomes) transduction methods [332], microfluidics has the ability to precisely control the physical force used and flow pattern to handle single cells. Yu et al. [333] reported that a cell transfection device relied on convective volume exchange between cells and surrounding fluid when an applied mechanical force generated cell deformation and reversible membrane fracture. The system was assessed using B-ALL-patient-derived T cells for CRISPR-based gene editing and CAR insertion with >60% efficiency and >80% cell viability. A hydroporation-based microfluidic vortex shedding platform was designed as an alternative to electroporation for CAR-T generation [334]. Cell membranes were gently permeabilized by oscillating fluidic forces generated by the vortex shedding flow. This device showed advantages in the ability to scale up large-scale CAR-T production, which is important for clinical applications. An acousto-electric microfluidic platform for dosage-controlled mRNA delivery was developed and used for the titration of CAR expression on T cells targeting B-ALL [335]. This platform could generate uniform CAR-T cells and be used to study the effect of CAR density on CAR-T cell functionality.

In general, microfluidic methods show advantages in low cost, high throughput, and high viability of recovered cells for T cell purification during CAR-T cell productions, while still facing challenges in scalability as large volumes of PB are needed for CAR-T therapy production (hundreds of ml to liters) [336]. The higher throughput in microfluidics can be secured by designing chips with a high number of parallel high-aspect ratio channels [337].

## 6. Summary and Conclusions

Blood disorders and diseases represent a significant global health problem. For many of these disorders, monitoring of the disease burden should be performed frequently and results delivered in real time to provide the appropriate treatment for possible impending acute disease. Microfluidics-based testing offers alternatives to standard methodologies, to secure such information. Microfluidics with various modes of operation have been developed for the isolation and detection of rare liquid biopsy markers (e.g., diseased cells, EVs, or circulating cell-free molecules) for diagnosis, prognosis, monitoring treatment response as well as fundamental characterization of the behavior of healthy and diseased cells. Microfluidics enable the high-throughput sorting of different blood components, allowing for the enrichment of specific subpopulations. In addition, these devices demonstrate the ability to measure the biophysical properties of blood cells to study pathologies such as anemia or thrombosis to provide information for monitoring early-stage abnormalities and disease progression. Blood cell interactions can be studied using vascular/tissue/organ-on-chip models to recreate vascular and BM microenvironments, providing physiologically relevant contexts for studying disease progression and predicting drug response [92,306,307]. Recent advances in EV isolation and characterization using microfluidics have made it possible to use EVs as circulating biomarkers for blood cancers and other disorders [338]. DNA/RNA analysis on-chip has provided the ability to secure information on gene mutation and expression at the single-cell level. Microfluidic assays for protein analysis have the potential to deliver fast, low-cost, POCT detection of blood abnormalities. For low-resource settings, microfluidics can deliver diagnostic or monitoring capabilities previously confined to large hospital settings; the consequence will be the translation from centralized laboratory testing directly to local clinics, reshaping global health equity in hematology. This will be facilitated by the recent integration of clinical testing with machine learning, which can enhance the information content of microfluidics in hematological diseases for both translational and basic research applications.

The rapid evolution of artificial intelligence (AI) and machine learning is positioning these tools as integral components of clinical testing workflows, including those for blood disorders and hematological diseases. AI-assisted cell type identification based on morphology and immunophenotyping will enable large volumes of high-resolution images to be processed more rapidly, directly supporting the diagnostic workload of pathologists. There is growing interest in integrating machine learning with microfluidic systems to enable automated data analysis, pattern recognition, and diagnostic classification. Recent studies have demonstrated the potential of image-processing algorithms and machine learning models to extract clinically relevant features from microfluidic imaging data, facilitating automated identification of blood cell phenotypes and other disease-associated markers [196]. Although the application of machine learning to microfluidic systems remains in its early stages, it has already demonstrated a promising trajectory. Key anticipated benefits include improved standardization of data analysis, greater degrees of automation, and meaningful gains in the speed and throughput of clinical testing, which are prerequisites for broader translation into routine diagnostic practice.

Microfluidics have emerged as transformative toolkits for the study of blood disorders, and the identification and enumeration of rare cells, MRD detection, and enabling the design and replication of mechanical and biochemical environments for the evaluation of blood cell biophysical behavior at the microscale. The advances of lab-on-a-chip technologies for flow-based disease modeling and clinical testing may offer translation from the research bench to the patient bedside in the future. To accomplish these goals, microfluidics must transition from academic proof-of-concept projects to scalable, manufacturable products. This will require scaling up manufacturing in a cost-effective and reproducible manner to improve the accessibility of these platform technologies into diverse settings. This can be realized by repurposing existing micro- and nanoscale manufacturing pipelines as those used for the CD, DVD, and Blu-ray industries for chip production and the use of plastic-based substrates [60,83,164,173,176,227]. Furthermore, the clinical validation of microfluidic tests in diverse settings and among diverse populations are needed before they take the center stage in testing.

## Figures and Tables

**Table 1:** Blood and bone marrow elements in healthy and diseased states.

Element	Disorder	Disorder Presentation in Blood or Bone Marrow	Normal Reference in Blood	Normal Reference in Bone Marrow
Red Blood Cells	Polycythemia	>6.1 × 10^6^/µL, Hb > 16.0 g/dL, and Hct > 49%	4.2–6.1 × 10^6^/µL, Hb 12–18 g/dL, Hct 37–49% [30]	Total 2.2 × 10^11^ 110 × 10^6^/µL * [31]
	Anemia	<4.2 × 10^6^/µL, Hb < 12 g/dL, Hct > 36%		
Platelets	Thrombocytopenia	<150 × 10^3^/µL	150–400 × 10^3^/μL [30]	1.5 × 10^11^ ** 75 × 10^6^/µL [31]
	Thrombocytosis	>450 × 10^3^/μL		
	Leukopenia	Mild: 1000–2000/μL; severe: <500/μL		
White Blood Cells	Leukocytosis	>11 × 10^3^ cells/µL >100 × 10^3^ cells/µL (hyperleukocytosis)	All nucleated cells: 4.0–10.8 × 10^3^/μL Neutrophils 40–73% Lymphocytes 19–48% Monocytes 0.4–10.0% Eosinophils 0–7.0% Basophils 0–2.0% [30]	Nucleated cells *: (0.8–1.5) × 10^12^ 500 × 10^6^/µL Granulocytes 57–86% Lymphocytes 9–27% (B-cell lineage 1–7%, T-cell lineage 8–20%) Monocytes 2–5.8% Megakaryocytes 0.05% Fibroblastic stromal cells < 0.2% Erythroid progenitors 4–16% Lymphoid progenitors 0.02–0.18% Myeloid progenitors 0.2–0.8% HSCs 0.01–0.2% [25,31]
	Non-Hodgkin Lymphoma	Cancer cells in lymph nodes. In bone marrow cancer cells: 0–50%; in blood abnormal lymphoma cells detected [32]		
	Hodgkin’s Lymphoma	Cancer cells in lymph nodes: Reed-Sternberg cells < 5% tumor cellularity, Hodgkin Reed-Sternberg cells (<2% tumor cellularity) [26,33,34]		
	AML	≥20% of myeloid lineage blasts in the blood or bone marrow [30]		
	CML	Chronic phase (blast cells < 10%), accelerated phase (blast cells 10–19%), and blast phase (blast cells > 20%) [30]		
	ALL	≥25% lymphoblasts in the bone marrow or peripheral blood [35]		
	CLL	Chronic phase (blast cells < 2% in blood, <5% in BM), accelerated phase (blast cells 10–19% in blood or BM), blast phase (blast cells ≥ 20% in PB or BM) [36]		
Plasma Cells	MGUS	Low or no blast count, serum M-protein level < 3 g/dL, free light chain ratio < 0.26, increase in λ light chain level	<5% blasts ~0.1% plasma cells [30]	<2% blasts <2% plasma cells [25]
	SMM	Circulating plasma cells in the blood, serum M-protein ≥ 3 g/dL		
	MM	Circulating plasma cells in the blood, M-protein ≥ 3 g/dL, ≥10% plasma cells in BM [35]		

Note: acronyms: Hb—hemoglobin; Hct—hematocrit; AML—acute myeloid leukemia; CML—chronic myeloid leukemia; ALL—acute lymphoid leukemia; CLL—chronic lymphoid leukemia; MGUS—monoclonal gammopathy of undetermined significance; SMM—smoldering multiple myeloma; MM—multiple myeloma; *—a reference standard assumes mass of 70 kg/154 lb individual, BM volume of 2 L; **—cell production per day [31].

**Table 2:** Material characteristics relevant to clinical and cell-based assays.

Material Characteristic	Material				
	Glass	Paper	PDMS	Flexdym	Thermoplastics (PMMA, COC, COP)
Bio- compatibility	Yes	Yes	Yes	Yes	Yes
Gas Permeability (Barrer) allows O_2_/CO_2_ gas exchange	None, <0.01	Porous, >1	Yes, ~500	Yes, 7.9–16	Very low, 0.05–5
Evaporation of solution	No	Yes	Yes	No	No
Elasticity, Young’s modulus (GPa)	None, 50–90	Low, 1–10	High, 0.001–0.003	Yes, 0.0012	Low, ~2.5–3.3
Surface chemistry and functionalization	Stable silane chemistry	Established and stable	Naturally hydrophobic; activated by oxygen plasma for bonding and hydrophilicity, not stable	Hydrophobic, activated by oxygen plasma to make hydrophilic, stable for a week	Naturally hydrophobic, activated by UV/ozone or oxygen plasma to make hydrophilic, stable for months
Optical transparency	Very good	Poor	Good	Good	Good
Fabrication	Photolithography, wet/dry etching. Complex and costly, low production rate	Wax printing, laser cutting, mass production possible	Soft lithography, rapid prototyping, easy but time-consuming molding, injection molding allows higher production rate	Injection, extrusion, roll-to-roll, hot embossing	PMMA: CO_2_-laser micromachining, injection molding, hot embossing, extrusion COC/COP: injection molding, hot embossing High production rate possible
Cost	High	Low	Moderate	Moderate/low	Moderate/low
Limitations	Brittle, expensive fabrication, no gas permeability	Poor mechanical stability	Absorbs molecules and drugs; swells in organic solvents	Slow process of device bonding	Low gas permeability, lack of flexibility

**Table 3:** Representative microfluidic assays for blood disorder testing.

Disorder	Microfluidic Model	Analysis	Citation
Sickle cell disease	Endothelium-on-a-chip with heme activation and flow adhesion assays	Adhesion of HbS RBCs to heme-activated human endothelial cells	[256]
	Computational fluid dynamics simulation of blood flow informed by microfluidic geometry (Carreau model; Murray’s law)—microfluidic platform geometry modeled in silico	Blood flow dynamics; RBC interactions and vaso-occlusion formation	[261]
	Microfluidic channels, object tracking with computational analysis	Blood flow resistance (viscosity, wall friction)	[262]
	Hypoxic microfluidic platform capable of inducing sickling/unsickling in flow; image analysis and dissipative particle dynamics simulations	Coupled HbS polymerization and RBC adhesion dynamics under hypoxia	[263]
Anemia, blood agglutination	Disposable microfluidic device with preloaded antibodies (anti-A, anti-B, anti-D); micro-slit agglutination readouts	Blood typing via agglutination; screening via differences in agglutination and density	[264]

## Data Availability

No new data were created or analyzed in this study. Data sharing is not applicable to this article.

## Abbreviations

2D	Two-dimensional
3D	Three-dimensional
aCSF	Artificial cerebrospinal fluid
AFM	Atomic force microscopy
ALCL	Anaplastic large cell lymphoma
ALL	Acute lymphoblastic leukemia
AMMoL	Acute myelomonocytic leukemia
AMoL	Acute monocytic leukemia
AML	Acute myeloid leukemia
APC	Antigen-presenting cells
APL	Acute promyelocyte leukemia
BM	Bone marrow
BMB	Bone marrow biopsy
BMA	Bone marrow aspirate
B-ALL	B-cell ALL
CAR	Chimeric antigen receptor
CAR-T	Chimeric antigen receptor T cell
CCD	Charge-coupled device
CLC	Circulating leukemia cells.
CLL	Chronic lymphocytic leukemia
CML	Chronic myeloid leukemia
CNS	Central nervous system
COC	Cyclic Olefin Copolymer
COP	Cyclic Olefin Polymer
CPC	Circulating plasma cells
CRISPR	Clustered regularly interspaced short palindromic repeats
CTC	Circulating tumor cells
CT	Computer tomography
DEP	Dielectrophoretic/dielectrophoresis
DLBCL	Diffuse large B-cell lymphoma
DLD	Deterministic lateral displacement
EV	Extracellular vesicle
ECM	Extracellular matrix
FC	Flow cytometer
FFPE	Formalin-fixed, paraffin-embedded
FISH	Fluorescence in situ hybridization
GOQD	graphene oxide quantum dot
Hb	Hemoglobin
Hct	Hematocrit
HL	Hodgkin lymphoma
Ig	Immunoglobulin
Ig/TR	immunoglobulin and T-cell receptor gene rearrangements
JMML	Juvenile myelomonocytic leukemia
LODs	Limits of detection
MANC	Multivalent aptamer nanoclimber
MCV	Mean corpuscular volume
MDS	Myelodysplastic syndrome
MFC	Multiparameter flow cytometry
MGUS	Monoclonal gammopathy of undetermined significance
MM	Multiple myeloma
MRD	Minimal residual disease
MRI	Magnetic resonance imaging
μSAAC	micropatterned super-porous absorbent array chip
NGS	Next-generation sequencing
NK	Natural killer
NHL	Non-Hodgkin lymphoma
OSB	Osteoblast
PB	Peripheral blood
PCL	Plasma cell leukemia
PCNSL	Primary central nervous system lymphoma
PDMS	Polydimethylsiloxane
PET	Positron emission tomography
POCT	Point-of-care testing
qPCR	Quantitative polymerase chain reaction
RBC	Red blood cell
SCD	Sickle cell disease
SMM	Smoldering multiple myeloma
TCR	T-cell receptor
TDT	Terminal deoxynucleotidyl transferase
T-ALL	T-cell ALL
VDJ/VJ	Variable, diversity and joining gene segments
vWF	Von Willebrand factor
WBC	White blood cells
