# Correction: Hu et al. Microfluidics for Blood Disorders and Hematological Disease Monitoring and Modeling. *Int. J. Mol. Sci.* 2026, *27*, 4581

**DOI:** 10.3390/ijms27209089

**Published:** 2026-10-14

**Authors:** Mengjia Hu, Nathan Henderson, Steven A. Soper, Malgorzata A. Witek

**Affiliations:** 1 Department of Chemistry, The University of Kansas, Lawrence, KS 66045, USA; m367h922@kumc.edu (M.H.); nhenderson4@kumc.edu (N.H.); 2 Center of BioModular Multiscale Systems for Precision Medicine, The University of Kansas, Lawrence, KS 66045, USA; 3 Kansas Institute for Precision Medicine, University of Kansas Medical Center, Kansas City, KS 66160, USA; 4 Department of Cancer Biology, University of Kansas Medical Center, Kansas City, KS 66160, USA; 5 Bioengineering Program, The University of Kansas, Lawrence, KS 66045, USA; 6 Department of Mechanical Engineering, The University of Kansas, Lawrence, KS 66045, USA

The following reference [69] has been retracted and should be removed from the original publication [1]:

69. Ouyang, D.; Ye, N.; Yang, K.; Wang, Y.; Hu, L.; Chao, S.; Toner, M.; Li, Y. Precision Isolation of Circulating Leukemia Cells in Chronic Myelogenous Leukemia Patients Using a Novel Microfluidic Device and Its Clinical Applications. *Cancers* **2023**, *15*, 5696.

With this correction, the order of some references has been adjusted accordingly. 

The authors state that the scientific conclusions are unaffected. The corrections were approved by the Academic Editor. The original publication has also been updated.
